# Rockefeller, the Flexner Report, and the American Medical Association: The Contentious Relationship Between Conventional Medicine and Homeopathy in America

**DOI:** 10.7759/cureus.87291

**Published:** 2025-07-04

**Authors:** Dana Ullman

**Affiliations:** 1 Family Medicine, Homeopathic Educational Services, Berkeley, USA

**Keywords:** consultation clause, flexner report, frederick t. gates, george h. simmons, homeopathy science, john d. rockefeller, john d. rockefeller jr, rockefeller institute for medical research, william osler

## Abstract

This article examines the ideological and institutional forces that led to the marginalization of homeopathy in American medicine, despite its popularity among prominent figures, including John D. Rockefeller. Drawing on five previously unpublished reports written for Rockefeller by his philanthropic and business advisor, Frederick T. Gates, the article reveals how these internal communications criticized homeopathy while shaping Rockefeller philanthropic foundations' policy. Using early 20th-century archival materials, it places the foundations' decisions within the broader context of changes in American medical education and regulation. The resulting funding decisions played a critical role in medical education reform and the rise of “scientific medicine.” The article also summarizes several strategies used by the American Medical Association (AMA) to marginalize homeopathy, including the “consultation clause” in its code of ethics, its collaboration in the writing of the Flexner Report, and its early advertising policies - an underexamined but significant factor in the AMA's consolidation of power and accumulation of financial resources.

## Introduction and background

Rockefeller’s use of and advocacy for homeopathy

John Davison Rockefeller (1839-1937) arguably had a more significant impact on 20th-century American medicine than any other individual, largely due to his philanthropic contributions to medical institutions, which totaled between $500 million and $1 billion in early 20th-century currency [1-4].

Raised by a pious mother who believed in tithing at least 10% of their income to charity, Rockefeller quickly became the most significant philanthropist in American history. Despite the substantial impact of Rockefeller’s financial contributions on American medicine, there has been, at best, only one book, *Rockefeller Medicine Men*, that provides some in-depth historical analysis of this significant influence and its historical context. The book is authored by a public health professor who acknowledged having limited knowledge or experience with homeopathy [5]. While objectivity is critical, expertise and experience in the subject can provide deeper and more valuable insight.

Even though John D. Rockefeller Sr., America’s richest man and first billionaire, provided substantial financial support to conventional medical schools and institutions, his personal medical care was supervised by doctors specializing in homeopathic medicine, a completely different type and style of treatment.

The word “homeopathy” derives from the Greek *homoios (*“similar”) and *pathos* (“suffering” or “disease”). It is a system of medicine developed in the early 19th century, based on the idea that a substance causing symptoms in a healthy person may be used to treat similar symptoms in someone who is ill. Although this pharmacological system, known as "the principle of similars," may initially seem confusing, its logic lies in the recognition that the body's symptoms are part of its evolved strategies for defense and survival. Emil von Behring, the first Nobel Prize recipient in medicine, for his discovery of the diphtheria vaccine, and later the tetanus vaccine, acknowledged that he drew inspiration for this discovery from homeopathic medicine [6].

Rockefeller, who lived to the age of 97, relied on homeopathic physicians for the last 50 years of his life. His first homeopath was Myra King Merrick, MD (1825-1899), the first woman doctor in Ohio, who helped establish the Cleveland Homeopathic Hospital College for Women in 1868. Dr. Merrick specialized in obstetrics and delivered John D. Rockefeller Jr. When Rockefeller moved to New York, he received homeopathic treatment from another female physician, Mary Belle Brown, MD (1850-1924), who graduated from the New York Homeopathic Medical College in 1879, taught at that hospital for 27 years, and served as dean from 1890 to 1898, as described by Kirschmann in *A Vital Force*, pages 1-2 [7].

In the 1890s, Hamilton Fisk Biggar, MD (1839-1926), began providing homeopathic care to the Rockefeller family. Biggar, J.D. Rockefeller’s long-time neighbor and golfing partner, became a frequent travel companion to Rockefeller during much of the first three decades of the 20th century.

Beginning in 1889 and persisting for several years, Rockefeller experienced significant fatigue and depression, partly due to his excessive workload, as described by Chernow in *Titan*, page 319 [1]. Biggar warned Rockefeller that he was on the verge of a breakdown and could potentially die, as described by Chernow in *Titan*, page 321 [1]. He began treating Rockefeller with homeopathic medicines in the early 1890s and recommended more vacation time. These measures helped restore Rockefeller to good health, and he lived for another 48 years.

Rockefeller suffered from alopecia, leading to the loss of all his body hair. Despite consulting various specialists, he received ongoing care exclusively from Biggar, which significantly improved his overall health, although his hair did not regrow [8].

According to Kirschmann in *A Vital Force*, page 46 [7], not only did Rockefeller use homeopathic physicians, but all of the Standard Oil families sought homeopathic care, primarily with Dr. Merrick. Merrick was highly respected by both homeopathic and conventional physicians, and it was rumored that various conventional obstetricians secretly consulted with her on their more complex cases [9].

Among the cultural elite of America, Rockefeller was not the only one who had a particular interest in and advocated for homeopathy. Esteemed medical historian William Rothstein wrote in his seminal 19th-century book on American medicine that “early American homeopaths were all well-educated and cultured physicians ... and manifested an erudition rarely found in regular medical journals of the period,” as described by Rothstein in *American Physicians*, page 160 [10]. Further, he quotes an editor of a leading conventional medical journal who begrudgingly admitted that many homeopaths were “persons of the highest respectability and moral worth.”

Many other members of the American ultra-rich were advocates of homeopathy, including George Westinghouse (founder of Westinghouse), Henry J. Heinz (founder of Heinz), George Eastman (founder of Eastman Kodak), Joseph Wharton (cofounder of Bethlehem Steel and founder of the Wharton School of Business at the University of Pennsylvania), Charles Kettering (founder of Delco and vice president of General Motors), and Hiram Sibley (founder of Western Union), as described by Ullman in *Homeopathic Revolution*, pages 229-51 [11].

In his Pulitzer Prize-winning book, *The Social Transformation of American Medicine*, page 97, Paul Starr explained why homeopathy attracted so many of the educated and wealthy classes: “Because homeopathy was simultaneously philosophical and experimental, it seemed to many people to be more rather than less scientific than orthodox medicine” [12].

In the early 20th century, homeopathic physicians often had very prosperous practices because they commonly treated many of America’s wealthiest and most educated elite, while their conventional medical colleagues often struggled financially.

Amid growing tensions between competing medical philosophies, reformers began calling for a standardized overhaul of medical training-a movement that would soon enlist the talents of Abraham Flexner.

Before Abraham Flexner was asked to author his analysis of medical education, he was known as a critic of rote memorization and a champion of progressive education models, including those of John Dewey. As historian Thomas Duffy notes, Flexner believed students learned best through active problem-solving and experiential learning, not passive information recall. This educational philosophy offers important context for understanding his later role in shaping medical training through the Flexner Report.

While Rockefeller’s personal preference for homeopathy contrasts sharply with his foundation's eventual rejection of it, this article moves beyond that paradox to examine a broader question: How did powerful institutional alliances, including the AMA, the Rockefeller foundations, and Abraham Flexner, shape the meaning of "scientific medicine" and marginalize homeopathy? Drawing on previously unpublished primary sources, it aims to clarify overlooked terms, reexamine forgotten evidence, and explore how institutional influence redefined medical legitimacy in the early 20th century.

This context makes it especially significant that this article presents five previously unpublished private reports written exclusively for Rockefeller by his philanthropic advisor, Frederick T. Gates. These reports harshly criticized homeopathy and its founder, Dr. Samuel Hahnemann, while praising Johns Hopkins professor Dr. William Osler. Gates appeared unaware that Osler had publicly expressed respect for homeopathy and its practitioners. Had these reports been made public, their inaccuracies could have been readily challenged.

These reports, along with other ideological and institutional factors, contributed to the Rockefeller Foundation's decision to withdraw support from any medical institution bearing the word “homeopathic” in its name. This occurred despite Rockefeller’s personal preference and his foundation's distributing between $500 million and $1 billion to medical institutions during the early 20th century.

The American Medical Association (AMA) was founded in 1847, two years after the establishment of the American Institute of Homeopathy, and was, in part, created to counter this leading competitor, as described by Rothstein in *American Physicians*, page 170 [10]. To marginalize homeopathy, the AMA implemented a policy known as the "consultation clause," which forbade orthodox physicians from associating with homeopaths. When these efforts proved insufficient, it covertly collaborated with Abraham Flexner in drafting the Flexner Report, an aspect rarely explored in medical history literature. The report’s widespread influence was made possible through substantial funding from the Rockefeller Foundation, which enabled its recommendations to reshape American medicine.

The article also explores the AMA’s early advertising policies, a rarely discussed but significant revenue source that helped fuel the organization’s expansion and dominance in American medicine.

The contentious relationship between homeopathy and allopathy in the 19th century

To better understand the antagonism J.D. Rockefeller faced with his advocacy for homeopathy, it is helpful to review some of the contentious history between these two leading schools of medical thought and practice. A trite but often true statement is that history is written by the victors. Consequently, our medical history textbooks today tend to glorify “scientific medicine” and assert that homeopathic medicine was an aberrant pseudoscientific treatment that acted merely as a placebo, its usage waning as “scientific medicine” gained greater acceptance and popularity. This historical review presents a reinterpretation of medical history that challenges conventional narratives. While it does not claim greater accuracy, it aims to provide additional context for a better understanding of the complexities of history.

The founder of homeopathic medicine, Samuel Hahnemann, MD (1755-1843), had a contentious relationship with the conventional physicians and apothecaries (pharmacists) of his time. Dr. Hahnemann was highly critical of most conventional medical treatments of the late 18th and early 19th centuries, particularly bloodletting, the use of mercury and arsenic, and the routine application of leeches, as described by Coulter in *Divided Legacy*, pages 101-39 [13]. Although Hahnemann’s views were met with hostility during his lifetime, modern medical historians have recognized the validity of many of his critiques, as described by Rothstein in *American Physicians*, page 170 [10].

Furthermore, because apothecaries were often paid based on the quantity of each drug they prescribed, Hahnemann and his fellow homeopaths were widely despised [14]. Not only did they prescribe extremely low doses of medicinal agents, but they also often recommended just one drug at a time. By contrast, conventional physicians of that era typically prescribed multiple drugs in high doses. Is it any wonder that the economic incentives in medicine, which promote prescribing more drugs in higher doses, have led to ongoing contentious issues?

Because Hahnemann did not trust apothecaries to properly prepare the medicines he prescribed, he sometimes made them himself. As a well-versed chemist and the author of the popular Pharmaceutical Lexicon, Hahnemann was highly skilled in his craft. However, frequent arrests by local authorities forced him to relocate and change his residence twenty-three times within 15 years [14]. In the 19th century, apothecaries wielded significant enforcement powers over their competitors, much as they do today.

The relationship between Dr. Hahnemann and homeopathy with conventional medicine initially began on good terms. Christoph Wilhelm Hufeland, MD (1762-1836), Germany’s most renowned and respected physician of his time, was as famous as Goethe and Schiller in the early 19th century. As the editor of Germany’s leading medical journal, Journal der Praktischen Arznei und Wundarzneikunde, Hufeland published some of Hahnemann’s writings and held him in high regard. In 1832, Hahnemann was even granted honorary membership in the Medical Society of the County of New York, as described by Kaufman in *Homeopathy in America*, page 32 [15]. However, a few years later, the society rescinded his membership when they determined that homeopathy’s growth posed an “ideological and financial threat” [16]. These perceived threats intensified the contentious relationship between the two systems of medicine.

In 1844, the American Institute of Homeopathy became the first national medical society established in the United States. In 1847, the AMA was founded, partly to curb the growth of homeopathy, as stated in its charter, as described by Rothstein in *American Physicians*, page 170 [10].

To undermine its primary competition, the AMA discouraged any association or communication with homeopathic doctors. The AMA established a code of ethics that contained a clause known as the “consultation clause.” This clause stated that if orthodox physicians merely consulted a homeopath or any other “non-regular” practitioner, they would lose their membership in the AMA [17]. In some states, losing membership in the local medical society also meant losing the license to practice medicine. To counter these restrictions, homeopaths established their own local societies and medical boards.

In the mid- and late-1800s, American physicians were rarely held accountable or reprimanded by their peers for medical malpractice, alcohol or drug abuse, sexual relations with patients, or any other unprofessional behavior. Yet almost any collaboration with a homeopath was considered actionable.

One New York physician claimed that the only ethical code regularly enforced was against those who consorted with or referred patients to homeopaths, as described by Coulter in *Divided Legacy*, page 208 [13]. A Connecticut doctor was expelled from his local medical society for consulting with a homeopath, his own wife. A New York physician was expelled for purchasing milk sugar from a homeopathic pharmacy. Joseph K. Barnes, the surgeon general of the United States, was denounced for aiding in the treatment of Secretary of State William Seward on the night Seward was stabbed and President Lincoln was shot, simply because Seward’s physician was a homeopath, as described by Coulter in *Divided Legacy*, pages 208-9 [13].

The AMA sought to expel homeopathic physicians from all local medical societies, as described by Coulter in *Divided Legacy*, page 199 [13]. Every state successfully purged homeopaths from its societies, except Massachusetts and New York. Homeopathy was so popular among the political, corporate, and cultural elite in these two states that the AMA temporarily permitted an exception, on the condition that the societies did not admit any new homeopathic members. In 1871, the Massachusetts Medical Society expelled its remaining eight physicians for the “crime” of being homeopaths.

Martin Kaufman, author of *Homeopathy in America: The Rise and Fall of a Medical Heresy*, observed that the media sympathized with the homeopaths. He quoted the editor of *The Boston Post*, who described the trial as nothing short of an “inquisition.” The defendants were denied the right to challenge witnesses, the benefit of legal counsel, and the power to exclude jurors who had previously displayed bias against them. Furthermore, the medical board acted as both prosecutor and jury, which seemed to make a mockery of the American legal system, as described by Kaufman in *Homeopathy in America*, page 84 [15].

Newspapers of the time were critical of the actions of the AMA and the Massachusetts Medical Society. The Boston Daily Journal declared, “It seems pitiable that these men, nearly all of whom have been educated at Harvard University and pronounced thoroughly competent to practice medicine, and who have so long enjoyed the confidence and respect of the community, should be consigned to so fearful a doom,” as described by Kaufman in *Homeopathy in America*, page 80 [15].

In 1882, the AMA expelled the entire New York State Medical Society from the national organization after the state society passed a resolution recognizing all properly graduated doctors, including homeopathic physicians. In response, the AMA established a competing state organization: the New York State Medical Association. These two separate state medical organizations persisted for 22 years, until 1906.

Henry Bowditch, MD, professor of clinical medicine at Harvard and a former president, vice president, and secretary of the AMA, protested the AMA’s actions, accusing it of destroying “life-long friendships” and creating dissension within the national organization [18].

The subject was editorialized by The New York Times, which stated: “The AMA says that if a patient’s life cannot be saved except by such a consultation, then the patient must die, and no doctor who will allow a homeopathist to help him can be recognized by the Association,” as described by Coulter in *Divided Legacy*, page 314 [13].

Despite the AMA’s efforts to discredit homeopathy, this alternative system of medicine continued to gain acceptance and respect among the American public, particularly its most esteemed citizens. There were more than 100 American homeopathic hospitals, around 1000 homeopathic pharmacies, and at least 20 homeopathic medical schools, including Boston University, the University of Michigan, the University of Minnesota, Hahnemann Medical College (in Philadelphia), the New York Homeopathic Medical College, and even the University of Iowa. Moreover, many of the American cultural elite, including prominent literary figures such as Henry James, William James, Washington Irving, Louisa May Alcott, Harriet Beecher Stowe, Emily Dickinson, Nathaniel Hawthorne, and Henry Wadsworth Longfellow, along with leading religious leaders and businessmen, expressed interest in and endorsed this “new medicine” as described by Ullman in *Homeopathic Revolution*, pages 63-74 [11].

The global appeal of homeopathy was also evident among powerful political figures, including royalty. Despite the significant power and influence wielded by royals and czars, they commonly encountered unwavering antagonism toward homeopathic medicine from both physicians and apothecaries, the drug manufacturers of that era. Dr. Carl Frantz Dominik von Villers, a German physician trained in conventional medicine who later practiced homeopathy in St. Petersburg, observed that the opposition to homeopathy among Russian physicians and apothecaries was so strong that even the czar’s considerable powers were ineffective in breaking down “the Chinese wall by which the medical hierarchy surrounds its domain” as described by Ullman in *Homeopathic Revolution*, page 279 [11].

## Review

Methods

This analysis draws on seldom-cited archival materials, including five previously unpublished reports written exclusively for John D. Rockefeller by his business and philanthropic adviser, Frederick T. Gates. These primary source documents are examined alongside AMA records, contemporaneous publications, and historical commentary to assess the ideological and institutional factors that influenced Rockefeller’s philanthropic choices and the marginalization of homeopathy in American medicine.

Results

In 1901, the AMA removed the consultation clause from its code of ethics, but this change was not due to a newfound acceptance of homeopathy. Homeopaths were allowed to join the AMA only if they denounced homeopathy and agreed not to use it in their practice, as described by Coulter in *Divided Legacy*, page 430 [13]. The AMA then devised new strategies to eliminate this competitive system of medicine.

George H. Simmons, MD, and the New AMA

In 1899, George H. Simmons, MD, assumed the roles of general secretary and manager of the AMA. He also became the editor of the Journal of the American Medical Association (JAMA), holding these positions for 25 years. At that time, JAMA’s advertising revenue was only $34,000 per year.

Simmons developed a reputation as a medical reformer and an advocate for various preventive, nonmedical strategies to improve public health, including child labor laws, minimum wage and working hours for both men and women, occupational safety, and the regulation of potentially dangerous chemicals, as described by Swenson in *Disorder*, page 39 [19]. Ultimately, one of Simmons’ long-time contributions to the AMA and to the medical profession was his starting of the medical specialty journals in the “Archive” series, including Internal Medicine, Surgery, Pediatrics, and others [20].

Of particular importance, Simmons devised a clever series of ideas that significantly increased funds for the organization. His plan fostered a deep and long-standing collaboration between various drug companies and the AMA. The AMA also introduced new guidelines for accepting advertisements in its journal [21].

Simmons proposed that the AMA establish the Council on Pharmacy and Chemistry to regulate the acceptance of drugs, provided there was a reliable means to verify the purity and strength of their ingredients. He also asserted that any advertisement in JAMA would imply AMA approval of the company and its drug, thereby enabling the AMA to charge higher advertising fees, as described by Swenson in *Disorder*, page 61 [19].

At the turn of the 20th century, American medicine was in a dire state. Corruption in medical commerce was rampant, as it was in many other sectors of American business at the time. Simmons claimed that drug manufacturers in England, France, and Germany created inferior product lines exclusively for the more open American market, where “the standard and quality of drugs have been left entirely to the manufacturers’ honor,” as described by Swenson in *Disorder*, page 61 [19].

Simmons referred to the industry’s “patent medicines” and “nostrums” of unknown composition as “a curse to our profession.” Because medical journals depended on advertisements, Simmons lamented that there was “no statement too silly, no claim too extravagant, and no falsehood too brazen” to be published in an advertisement [22].

To advertise in the new *JAMA*, the AMA did not require drug companies to provide evidence of efficacy or safety, but it did establish other guidelines that ensured important transparency for each drug being advertised. The emerging drug industry was encouraged by the fact that drug manufacturers were not required to provide independent evidence of the efficacy or safety of their products. The new advertising standards introduced consumer-friendly features while encouraging drug companies to focus their advertising budgets on the medical association’s magazines. The standards were as follows: (1) the AMA required drug companies to disclose all the ingredients in their medicines. During the late 1800s, many companies used secret formulas, but this consumer-friendly guideline mandated transparency. (2) The AMA discouraged physicians from advertising, and medical journals were pressured not to run advertisements for drugs that were marketed directly to the public. This policy forced pharmaceutical companies to restrict their advertising to medical journals instead of targeting the public. Consequently, the advertising revenue of the AMA and state and local medical societies increased significantly [23,24]. (3) In addition to the written rules, there were unwritten guidelines. Wallace Abbott, the owner of Abbott Laboratories, claimed that Simmons demanded “shakedown money” to secure the AMA’s seal of acceptance, enabling the company to advertise in AMA publications, as described by Davidson, *A Century of Homeopaths*, page 188 [25,26]. By charging exceptionally high advertising fees, Simmons leveraged the pharmaceutical companies’ need to advertise by insisting that they do so in various local, regional, and national publications.

Ultimately, it seems that these new drug advertising practices led to a formal and significantly lucrative relationship between the pharmaceutical industry and the AMA. However, some pharmaceutical companies were still able to circumvent the guidelines.

For example, rather than paying these high fees, Abbott hired an investigator to research Simmons’s background. The investigation revealed that Simmons had claimed to have obtained his medical degree from a hospital in Ireland that did not confer degrees. It also uncovered that Simmons had attended a homeopathic medical school in Chicago and practiced homeopathy for a decade. During this time, he actively advertised his practice, an act strongly discouraged by the AMA both before and after Simmons became the head of the organization. One of Simmons’s former wives provided an affidavit confirming the accuracy of this information. Shortly thereafter, Abbott Laboratories was permitted to advertise its products in AMA publications.

Simmons’s actions, regardless of their legality or ethics, significantly benefited the AMA. JAMA’s advertising revenue increased from $33,760 in 1899 to $150,000 in 1909, as described by Starr in *Social Transformation*, page 134 [12]. In 1900, the AMA had only 8,000 members; by 1910, its membership had grown to over 70,000 [27]. These figures marked only the beginning of the association’s growth.

Another respected physician who was highly critical of Simmons was Frank Lydston, MD, a urologist and transplant surgeon. Like Abbott, Lydston uncovered a substantial amount of unethical conduct by Simmons. Lydston was particularly alarmed by the authoritarian powers that Simmons had assumed for himself. He even took Simmons and the AMA Board of Directors to court, arguing that AMA members had never elected the board. After a five-year legal battle, the Illinois Supreme Court upheld an earlier appellate court ruling that ordered the removal of the AMA’s officers [28].

Lydston also provided evidence from Simmons’s writings in medical journals, demonstrating that Simmons claimed to know all there was to know about “regular medicine” (conventional medicine). However, Lydston referred to him as “an egotistic, boastful homeopath, mind you, not a modest one” [29].

If these controversies were not enough, Simmons divorced his first wife in 1892, but in 1917, she sought to have the divorce annulled. She presented evidence of the fraudulent use of her signature and alleged that Dr. Simmons had her undergo several surgeries, which led her to a morphine addiction, leading her to believe she was going insane, and which would ultimately lead to her being committed to an insane asylum [30]. The trial was so sensationalized that it was theorized to have inspired Patrick Hamilton to write the highly successful Broadway play Angel Street, which later became the classic film Gaslight, starring Ingrid Bergman. It is from this play and film that the term “gaslight” gained popularity, as described by Davidson in *A Century of Homeopaths*, page 188 [25].

In 1924, after ongoing controversies, Simmons was compelled to resign. His successor, Morris Fishbein, MD, noted that Simmons burned all his personal files after being forced out of his position at the AMA [31]. None of these controversies was mentioned in JAMA, nor were they referenced at a banquet held in Simmons’s honor in 1923 [32].

Simmons warned his successor that working with pharmaceutical manufacturers was “about the same as Faust trying to make a deal with Mephistopheles.” However, Fishbein often boasted about his strong relationships with prominent drug company representatives, particularly Elmer Bobst of Hoffmann-La Roche, as described by Swenson in *Disorder*, pages 432-3 [19].

Despite the significant impact of Simmons’s work in increasing the AMA’s membership and advertising revenues, few prominent books on the history of American medicine provide details about the life of Dr. Simmons. Chernow’s extensive biography of Rockefeller fails even to mention Simmons, let alone his influence on American medicine at the time. Similarly, the Rockefeller Archives Centre, which holds extensive information about the Rockefeller family, contains virtually no records or correspondence relating to Simmons.

The AMA was in a vastly different position in 1899 when Simmons began his work, compared to its state and status in 1924 when he left. Early in his tenure at the AMA, Simmons identified two major challenges facing conventional physicians and the organization. First, there was an oversupply of conventional doctors, and second, there was significant competition from various forms of “quackery,” including homeopaths, osteopaths, naturopaths, botanical practitioners, and eclectic physicians.

During the 1903 AMA annual convention, Joseph N. McCormack, MD, a Kentucky physician and leading force behind the reorganization of the AMA, attempted to persuade the organization to explore the homeopathic principle of similars. This move was not entirely surprising. At this convention, he acknowledged, “We must admit that we have never fought the homeopath on matters of principles; we fought him because he came into the community and got the business,” as described by Kaufman in *Homeopathy in America*, page 158 [15].

However, in the early 20th century, Simmons and his colleagues at the AMA developed an important next-step strategy to reduce competition for conventional physicians.

The Carnegie Foundation’s Flexner Report and Its Impact on Medical Education

At the same time George Simmons took over the AMA, J. D. Rockefeller began planning what would later become the Rockefeller Institute for Medical Research (RIMR). Once Rockefeller established his Rockefeller Institute, he hoped other wealthy individuals would create comparable and competing research institutes, and some did. For example, Andrew Carnegie (1835-1919), who founded the Carnegie Steel Company (later U.S. Steel), also established an educational institute, the Carnegie Foundation for the Advancement of Teaching.

Henry S. Pritchett was hired to head the new Carnegie Foundation. Pritchett met with Arthur Bevan, MD, head of the AMA’s Council on Medical Education, to develop a plan to improve medical education. Ultimately, they came up with the idea of surveying American medical schools. The AMA could not sponsor the study due to perceived bias, so Pritchett suggested that the Carnegie Foundation conduct it. This way, the AMA could achieve its objectives without appearing to sponsor the report.

Pritchett hired Abraham Flexner to survey medical colleges and develop guidelines for a model medical school. Flexner was the brother of Dr. Simon Flexner, the first director of the RIMR, a pathologist known for advocating "scientific medicine" and criticizing sectarian approaches, including homeopathy. At the time, Abraham Flexner was an unemployed schoolmaster with no medical training or relevant background in the field. Because he had not publicly expressed a preference for either homeopathy or allopathy, he was considered an impartial evaluator of medical schools. However, a critical question remains: Was Abraham Flexner truly an objective reviewer of medical education?

Even before Abraham Flexner surveyed medical schools, the AMA and the Carnegie Foundation had predetermined agendas. Both organizations expressed concerns that America had too many doctors. Publications from both organizations used the same statistic: Germany had one doctor for every 2,000 people, while the United States had one doctor for every 568 people, as described by Flexner in Report on Medical Education, page 14 [33]. It is difficult to understand why having a surplus of doctors would be a problem for anyone other than doctors.

The AMA had an absolute abhorrence for the various “irregular” methods of medical treatment, such as homeopathy, osteopathy, chiropractic, eclectic, and naturopathy. If something could be done to reduce or eliminate these competitors, conventional or “regular” doctors would be in greater demand and earn a better living. The AMA and the Carnegie Foundation were also concerned about the existence of three women’s medical colleges. Flexner believed that women were less able to withstand the mental rigors of being a physician and claimed that women made better patients than doctors, as described by Brown in *Rockefeller Medicine Men*, page 149 [5].

Flexner frequently consulted with AMA leadership and even visited medical schools throughout the United States with Nathan Colwell, secretary of the AMA’s Council on Medical Education. Flexner did not have a similar association with homeopathic physicians or organizations. Prior to the survey, representatives of the American Institute of Homeopathy attempted to meet with Flexner, but there is no record of any such meeting taking place, suggesting that the homeopaths were treated differently from the conventional physicians.

Furthermore, six months before the Flexner Report was published, Pritchett wrote a memo to Bevan of the AMA: “In all this work of the examination of the medical schools, we have been hand in glove with you and your committee. When our report comes out, it is going to be ammunition in your hands. It is desirable, therefore, to maintain in the meantime a position which does not intimate an immediate connection between our two efforts” [34].

This statement, which might be considered a classic “smoking gun,” clearly indicates that Bevan wanted to keep the collaboration and conspiracy between the AMA and the Carnegie Foundation a secret.

If the quoted memo from the Carnegie Foundation to the AMA was not strong enough evidence of collusion, a later AMA publication bragged that Colwell “provided far more guidance in this famous survey than is generally known,” as described by Coulter in *Divided Legacy*, page 446 [13].

Interestingly, Flexner claimed in his memoirs that Colwell visited “many” medical schools, yet at another time, he claimed that it was hardly more than half a dozen. However, Morris Fishbein, who succeeded Simmons as the AMA’s general manager and as editor of JAMA, claimed that Colwell visited “every school” with Flexner, as described by Swenson in *Disorder*, page 300 [19].

Flexner’s survey of medical education in the United States and Canada required him to visit 155 medical colleges and 12 postgraduate schools in just 16 months. He spent less than a day at each school.

Bevan was so anxious to get this information from the Carnegie Foundation’s report to the medical community and the public that he asked Flexner and Pritchett to speak at the AMA meeting several months before the report was published. However, Pritchett did not want the public to know about the close ties between the AMA and the Carnegie report, as described by Brown in *Rockefeller Medicine Men*, page 152 [5]. Some historians simply and directly assert that the AMA “engineered” this report [35].

*JAMA* praised the Flexner Report, but numerous other conventional medical journals, such as the Medical Sentinel, American Medical Compound, Therapeutic Gazette, and the Hospital Bulletin, criticized it as being “hasty,” “full of errors,” “a monumental piece of impudence,” and “unfair to small and worthy schools,” as described by Hiatt in *Pharos*, pages 73-87 [36].

William Osler, MD, one of the most respected physicians in America and Europe of that era and one of the four founding professors of Johns Hopkins Hospital, who authored a seminal textbook published in 1892 and used for over 40 years, asserted that Flexner had a “very feeble grasp of medicine.” He added that there were so many errors in Flexner’s report that he could not determine whether “unfairness or ignorance” was more prevalent. Osler believed that, in both cases, gross injustice had been done, as described by Hiatt in *Pharos*, page 19 [36].

Osler was particularly critical of Flexner’s recommendation that medical school education focus on laboratory science rather than clinical science. Flexner insisted that medical schools should employ only full-time faculty specializing in laboratory research, rather than physicians who also maintained part-time medical practices.

In 1911, Osler wrote a stern letter to Ira Remsen, MD, president of Johns Hopkins University, declaring, “I cannot imagine anything more subversive to the highest ideal of the clinical school than to hand over our young men who are to be our best practitioners to a group of teachers who are ex officio out of touch with the conditions under which these young men will live” [37].

Harvey Cushing, MD, a neurosurgeon and pathologist, was another leader of American medicine who, along with Osler, criticized the primary recommendations of the Flexner Report. However, according to Dr. Thomas P. Duffy’s 2011 review, “The Flexner Report-100 Years Later,” both Osler’s and Cushing’s opinions “were hushed by the irresistible seduction of large sums of money” [38].

Flexner considered the Johns Hopkins School of Medicine the “model” for the highest quality of scientific medical education available at the time. Despite this, criticism of the Flexner Report should have been fatal. However, the report had significant financial and political backing, making it an unstoppable force that plowed through any opposition.

Ironically, as mentioned, Ira Remsen was the president of Johns Hopkins at the time. Remsen graduated from New York Homeopathic Medical College and Flower Hospital in 1865. Later, he taught chemistry at the same institution. Remsen founded the *American Chemical Journal*, which he edited from 1879 to 1914, and co-discovered the artificial sweetener saccharin, as described by Davidson in *A Century of Homeopaths*, pages 147-8 [25]. Any criticisms that homeopathic medical education lacked rigor should have been muted by these and many other leading 19th- and 20th-century physicians initially trained in homeopathic medical schools.

However, facts may not have been the primary motivating factor for the work of Flexner, Gates, and the AMA. Flexner’s attitude toward what was called “sectarian medicine” (also referred to as “irregular medicine”) was extremely antagonistic. The term “sectarian” not only referred to the fact that such treatments were different from and alternative to the conventional medicine of the day, but it also suggested that these treatment methods focused on the forces of nature and the doctor within every person, concepts that were anathema to conventional or regular physicians of the time.

Flexner maintained great disdain for chiropractors, referring to them as “unconscionable quacks” and to eclectic doctors as “drug mad." Eclectic doctors graduated from medical schools that taught conventional, herbal, and homeopathic medicine. It was both strange and ironic that Flexner was so critical of the eclectics, given that they integrated many conventional medical treatments alongside a host of other methods. By contrast, Flexner equated homeopathy with “dogma.” He wrote in his report, “The ebbing vitality of homeopathic schools is a striking demonstration of the incompatibility of science and dogma” [39].

It may be helpful to note that only a minority of educated homeopaths were able to sustain a clinical practice using solely homeopathic medicines. Instead, most homeopathic doctors were eclectic, typically employing homeopathic medicines as their primary treatment method while also utilizing various orthodox and herbal treatments. It could be argued that it was the orthodox physicians who practiced dogma, as they refused to consider using homeopathic medicines or many of the popular herbal remedies available at the time.

In the 19th century, the vast majority of homeopathic physicians were trained at highly respected medical schools, setting them apart from other sectarian medical groups, such as chiropractors, naturopaths, midwives, or Thomsonian practitioners. This rigorous medical training made homeopathic physicians an even greater threat to the conventional or “regular” medicine of that era.

Flexner showed little interest in producing an “objective” report on medical education. He argued that “scientific medicine” need not be “democratic” or cater to the public’s interest in alternatives to orthodox medicine. Furthermore, he contended that dissent signified ignorance or “blind faith” in “dogma,” as described by Flexner in *Report on Medical Education*, pages 156-61 [33].

Despite the varied and serious concerns about the Flexner Report, it had a profound impact on American medical education. Flexner recommended the closure of all women’s medical colleges, and shortly after his report, all but one were closed, including five of the seven Black medical colleges. The 22 homeopathic medical schools in existence in 1900 had dwindled to just two by 1923.

The naturopathic, eclectic, and chiropractic schools experienced a similar fate. All medical schools teaching homeopathy were forced to revise their curricula to align with the guidelines advocated by Flexner. These revisions mandated a shift to a biomedical approach, which significantly curtailed homeopathic training. Consequently, homeopathic medical schools began producing less proficient homeopaths, and by 1950, all schools teaching homeopathy had closed. Ultimately, the Flexner Report led to a smaller, wealthier, and more homogeneous group of doctors in terms of race, gender, and type of therapeutic practice.

The Founding of the Rockefeller Institute for Medical Research

In the mid-1890s, Rockefeller endeavored to establish a medical institute at the University of Chicago “that was neither allopath nor homeopath, but simply scientific in its investigations into medical science” [40]. However, he was dissuaded from doing so by his medical advisers, who argued that any support for homeopathy was inappropriate, as described by Nevins in *John D. Rockefeller*, page 263 [41].

In 1901, John D. Rockefeller established the RIMR with a $20,000 grant, followed by a $1 million gift. Although the RIMR was primarily funded by Rockefeller, the individual responsible for managing the institute and making decisions regarding grants was Frederick T. Gates. Gates, a Baptist clergyman, had previously served as the executive director of the American Baptist Education Society. Notably, Rockefeller was also a Baptist.

Rockefeller informed Gates that he did not have the time to manage his philanthropic endeavors and requested that Gates administer the institute. Gates insisted that the institute operate independently and not be affiliated with any university to maintain the appearance of impartiality. Aware that Rockefeller would not want his institute associated with any allopathic medical school, Gates concealed his support for orthodox medical institutions from Rockefeller.

Rockefeller was known for avoiding interference in the affairs of his philanthropic organizations or in the organizations that received grants. His biographer, Allan Nevins, observed that “he kept his hands entirely off the management,” as described by Nevins in *John D. Rockefeller*, page 648 [41]. While Rockefeller expressed interest in supporting homeopathic institutions, none of his foundations provided a single grant to any homeopathic organization or institution during the 20th century, despite his philanthropy exceeding $500 million. His son, John D. Rockefeller Jr., upheld this policy [2].

Nevins correctly asserted that Rockefeller’s financial contributions “resulted in nothing less than a revolution in medical education,” as described by Nevins in *John D. Rockefeller*, page 493 [41].

One theory for why Rockefeller did not exert greater influence to support his favored homeopathic institutions is that he believed philanthropists should refrain from using their grant money for self-serving purposes and should act in a disinterested, public-spirited manner, as described by Chernow in *Titan*, page 468 [1].

Swenson theorized that Rockefeller was compelled to demonstrate significant philanthropic efforts to “shine up Standard Oil’s public image,” which had been tarnished by Ida Tarbell’s highly critical articles in Collier’s as well as her 1904 book, *The History of the Standard Oil Company*, as described by Swenson in *Disorder*, page 181 [19]. The fact that the Department of Justice was prosecuting Standard Oil under the Sherman Antitrust Act was also believed to be a contributing factor.

Gates capitalized on this opportunity by strategically allocating the institute’s funding to avoid aligning with Rockefeller’s special interests, viewing homeopathic institutions in this light.

Notably, Gates, initially hired by Rockefeller in 1892, personally selected key personnel for Rockefeller foundations and institutions who shared his strong opposition to homeopathy. These individuals included: (1) Starr Murphy, J. D. Rockefeller’s attorney, who actively examined projects before donations were made, who was personally introduced to Rockefeller by Gates, as described by Chernow in *Titan*, page 477 [1]. (2) Simon Flexner, the first director of the RIMR, who later served on its Board of Trustees. (3) Abraham Flexner, author of the famed Flexner Report, who was later hired by Rockefeller’s General Education Board to support the proposed guidelines of the report.

In 1910, with $8 million in support from the RIMR, a new 60-bed hospital with a nine-bed isolation pavilion was established in New York City. This hospital’s top floor included four rooms reserved for the Rockefeller family, yet John D. Rockefeller never utilized them despite Gates’s persistent urging. Rockefeller favored homeopathic and osteopathic treatment for himself and much of his family, as described by Brown in *Rockefeller Medicine Men*, pages 109-10 [5].

In 1916, Rockefeller expressed profound consternation to his staff, declaring, “I am a homeopathist. I desire that homeopathists should have fair, courteous, and liberal treatment extended to them from all medical institutes to which we contribute.” Similarly, in 1919, when he allocated $45 million to the General Education Board, he instructed his son and his staff, “Homeopathic teaching should not be excluded; it should be provided for, the same as Allopathic,” as described by Coulter in *Divided Legacy*, page 314 [13].

Gates had retired from his full-time position with the RIMR by 1916, but he continued to serve on its board of trustees until his death in 1929. In his absence, Gates brought in others who shared his opposition to sectarian medicine, including William Welch, MD, the first dean of the Johns Hopkins School of Medicine and a professor of pathology. Around this time, Rockefeller’s son also assumed a much more active role.

J. D. Rockefeller Jr., commonly known as Junior, was the only son in his family, alongside three sisters. Junior worked for Standard Oil until 1910, after which he devoted himself to philanthropy. In 1913, he was elected president of the Rockefeller Foundation, a position held for 21 years. He continued his work with the Foundation until 1954. While Junior supported cooperation among different religions, Gates’s influence led him to oppose collaborative approaches between the various schools of medical thought and practice.

The renowned Rockefeller Archive Centre in Tarrytown, New York, is believed to house all known files on homeopathic medicine and Rockefeller’s homeopathic doctors. However, these files offer limited insight into the developments after 1920. In fact, the entire file on “homeopathy” has no signed letters from John D. Rockefeller after 1920, leaving one to wonder why, given his profound interest in the subject, particularly as he lived for another 17 years.

Ron Chernow, another biographer of Rockefeller, acknowledged that Gates refrained from expressing his strong views on homeopathy. He stated that Gates’s “real aim was to deliver a mortal blow to homeopaths-to shut their medical schools, expel them from medical societies, and strip them of hospital privileges-so as to clear the field for scientific medicine,” as described by Chernow in *Titan*, pages 478-9 [1].

Frederick Gates and John D. Rockefeller Jr. had similar views about medical treatment. They both disliked homeopathy and believed that “scientific medicine” was the future. Gates criticized Hahnemann, referring to him as “a little less than a lunatic,” as described by Chernow in *Titan*, page 566 [1], despite Hahnemann’s reputation as an intellectual giant, the author of a leading chemistry text, and a physician to the German aristocracy.

J. D. Rockefeller’s significant donations to homeopathic hospitals and colleges ceased entirely when Gates took over his philanthropy. John D. Rockefeller Jr. donated $537 million up until 1960, the year he passed away, yet he refused to support any institution with homeopathy in its title, as described by Chernow in *Titan*, page 566 [1].

Before Gates arrived, Rockefeller had not yet established his philanthropic foundations; however, there were numerous instances where he donated to various homeopathic institutions, including the Cleveland Homeopathic College.

Rockefeller instructed his lawyer, Starr J. Murphy, to follow up on his recommendations to support homeopathic colleges and hospitals. However, in 1912, Murphy instead enlisted Abraham Flexner to begin working with Rockefeller’s General Education Board to implement the recommendations of the Flexner Report, which offered a harsh critique of homeopathy [42].

In communications with the Rockefeller Institute of Medical Research, Simon Flexner stated that homeopaths and their institutions were being treated fairly, an assertion that could reasonably be considered misleading, if not inaccurate. He wrote, we "make no distinction and welcome to their staff qualified men irrespective of the school in which they have been trained," as described by Brown in *Rockefeller Medicine Men*, page 110 [5].

Nevertheless, Flexner made this statement shortly after meeting with representatives of the New York Homeopathic Medical College and Flower Hospital, where he told their representatives that those colleges and hospitals with “homeopathic” in their name would be considered “sectarian” and, therefore, unsuitable for funding, Murphy continued misinforming his boss, stating, “The discriminations which formerly were practiced against homeopathists are being constantly lessened,” as described by Brown in *Rockefeller Medicine Men*, page 110 [5].

Medical doctors who graduated from homeopathic medical schools were permitted to join the AMA, but only if they agreed not to identify as homeopathic physicians. The complete lack of funding received by homeopathic institutions was evidence of the intense hostility displayed by representatives of the Rockefeller Foundation toward them.

In 1910, the Medical World magazine claimed that “medical bigotry is on the decline,” as described by Brown in *Rockefeller Medicine Men*, page 166 [5]. However, this claim can only be considered true in word, not in deed.

The Flexner Report’s Impact on Rockefeller’s Philanthropy

In 1912, shortly after the Flexner Report was published, Abraham Flexner was employed at Rockefeller’s General Education Board, where he worked tirelessly to provide substantial grants that helped to actualize the “reforms” recommended in his report. Flexner remained employed at this Rockefeller organization until 1928, as described by Chernow in *Titan*, page 404 [1].

The grant-giving organization provided at least $180 million from 1910 to 1930. Frederick Gates and Abraham and Simon Flexner managed the funds. This funding was used to promote and enforce the acceptance of the Flexner Report. There is no evidence that any homeopathic college or hospital received financial support [9].

This work, unnoticed by many, played a crucial role in shutting down the homeopathic and eclectic medical schools.

Before Gates created the various Rockefeller foundations, Dr. Biggar, Rockefeller’s homeopath, successfully obtained Rockefeller’s land donation and funds to construct the Cleveland Homeopathic Hospital College. Rockefeller briefly served as vice president before Gates took over the RIMR, as described by Kaufman in *Homeopathy in America*, page 146 [15]. However, once Gates created the Rockefeller grant-giving institutions, he ensured that no funding would be allocated to homeopathic institutions.

Harry Simms, who was Rockefeller’s secretary and later married a Rockefeller relative, served on the Board of Trustees of Cleveland Homeopathic Hospital College in 1922. He acted as chairman of the board from 1936 to 1956. Despite his efforts, even Simms was unable to secure any additional funding for the college after its construction, as described by Robins in *Copeland's Cure*, page 76 [43].

As medical schools and hospitals relied on governmental or philanthropic aid to sustain them, it became evident that the absence of funding from the Rockefeller Foundation for homeopathic schools and hospitals would ultimately lead to their downfall. That is precisely what occurred.

In their pursuit of funding, homeopathic institutions gradually removed the word “homeopathic” from their names and reduced the amount of training provided in this discipline. This shift led to the production of physicians who were inadequately trained in the homeopathic method.

The implementation of new medical licensing exams forced homeopathic schools to concentrate more on basic medical science, even if the content taught was not clinically relevant. Consequently, they were forced to sacrifice the in-depth study of the science and art of homeopathic medicine.

The AMA implemented a new strategy to promote what was described as “scientific medicine,” which led to the decline and eventual elimination of homeopathic and eclectic schools. The AMA’s Council on Medical Education established guidelines to evaluate schools based on specific standards aligned with biomedical research and the conventional medical model of treatment, which were subsequently used to grade the schools.

Every school was required to procure new and expensive medical equipment. However, this equipment was more suited to orthodox medical treatment than to homeopathic care. Furthermore, the Rockefeller Foundation exclusively supported orthodox medical schools in acquiring this new equipment. Consequently, homeopathic colleges and their students were placed at a considerable disadvantage.

Homeopaths fought diligently to gain recognition in the medical field. In 1894, the Association of American Medical Colleges officially recognized that homeopathic medical colleges were as effective in teaching medicine as conventional medical colleges, 16 years prior to the Flexner Report. The Association even passed a bylaw permitting homeopathic graduates to be admitted to advanced standing in orthodox medical colleges and to receive credit for all courses, except those solely dedicated to homeopathic clinical training [44].

According to a survey published by JAMA, homeopathic medical schools provided high-quality medical education. Surprisingly, the survey revealed that between 1900 and 1905, graduates of homeopathic medical schools had a lower failure rate on state medical board examinations than graduates of conventional medical schools. The failure rate for homeopathic graduates was 9%, compared to 16% for conventional medical graduates. Similarly, in 1909, the difference was 14.3% versus 16.2%, as described by Brown in *Rockefeller Medicine Men*, page 126 [5].

Flexner and Gates strongly contended that only the larger medical schools should continue to exist. However, statistics from 1912 show that larger medical schools did not necessarily have a higher percentage of students who passed the state licensing examination, as described by Coulter in *Divided Legacy*, page 463 [13]. Furthermore, Boston University, a homeopathic school, had a failure rate of only 3.3%, while Harvard’s failure rate was 11.8%.

When Gates first met Rockefeller, he was a devout Baptist pastor. However, shortly thereafter, Gates began reading the Bible more critically and appeared to have converted from his Baptist faith to what could be described as the religion of “scientific medicine,” as described by Chernow in *Titan*, pages 478-9 [1].

Rockefeller was once asked if he ever studied medicine. He responded, “No, not much, but I have studied doctors.” Indeed, he had, and perhaps that is why he always preferred homeopathic treatment for himself, and why he even outlived his homeopath, who only lived to be 87. Rockefeller’s next and last homeopath, Alonzo Eugene Austin, MD, was, like his predecessors, a devoted practitioner of classical Hahnemannian homeopathy.

Rockefeller described homeopathy as a “progressive and aggressive step” in medicine, as described by Coulter in *Divided Legacy*, page 463 [13]. Unfortunately, homeopathy could not withstand the power and influence of significant financial resources working against it.

Gates’s Influence on Rockefeller’s Understanding of Medicine

To date, no historian or biographer has written in detail about the five private reports that Frederick T. Gates prepared for J. D. Rockefeller, which influenced Rockefeller’s shift in support from homeopathy to “scientific medicine.” Only Rockefeller’s biographer, Ron Chernow, made a passing reference to one of these five reports where Gates criticized Rockefeller’s best friend, neighbor, and personal homeopath, Dr. Hamilton F. Biggar, as described by Chernow in *Titan*, pages 478-9 [1]. All five original reports written by Frederick T. Gates for John D. Rockefeller are provided in the Appendix.

Shortly after the publication of the Flexner Report in 1910, Gates began writing a series of reports on homeopathy. He chose not to publish these reports, as he admitted in a letter to J. D. Rockefeller dated 27 April 1911 (see Appendix). It is reasonable to assume that Gates’s intention in writing these five reports was to secretly influence Rockefeller’s thinking without having to face any scrutiny. Additionally, some of the medical information that informed Gates’s thinking was not well supported, not just by today’s standards but by what was known at that time.

In 1911, Gates wrote these five short reports titled “Notes on Homeopathy” (see Appendix). In his first report to Rockefeller, he expressed great admiration for Dr. William Osler. Specifically, Gates wrote: “In the first place, I have read the works of Dr Osler, who stands now, I believe, as the greatest modern physician. It was the reading of his works that led me to suggest the founding of the Rockefeller Institute.”

This assertion is remarkable for many reasons. First, Osler had genuine respect for homeopathy. In his 1905 farewell address to the American medical profession, he said:

"It is not as if our homeopathic brothers are asleep: far from it, they are awake-many of them at any rate-to the importance of the scientific study of disease. ... It is distressing to think that so many good men live isolated, in a measure, from the great body of the profession. The original grievous mistake was ours-to quarrel with our brothers over infinitesimals was a most unwise and stupid thing to do" [45].

Shortly after this, Osler spoke at a conference of nurses and proclaimed, “No one will deny that as many patients recover under homeopathic treatment as recover under any form of treatment,” as quoted in Robins's *Copeland's Cure*, page 59 [43].

Time magazine reported that, before Osler died in 1919, he expressed even greater support for homeopathy and its founder, asserting that “No individual has done more good to the medical profession than Samuel Hahnemann” [46].

The quotes above are further supported by the fact that two of Osler’s favorite treatments were “Nux vomica and hope” [47]. Nux vomica is a fruit of the strychnine tree, which is poisonous except in homeopathic doses. It is well known that Nux vomica is a popular homeopathic drug.

Despite these seemingly strong statements, it should be noted that Osler was a leading advocate of “therapeutic nihilism,” the belief that many medical treatments are ineffective, some are harmful, and that the body should be allowed to heal itself. Although Osler may have had some positive experiences with homeopathy, his appreciation for Hahnemann and homeopathy may have stemmed more from their focus on allowing the body to heal itself. Furthermore, Osler thanked Hahnemann and homeopathy for their critique of the common practice of polypharmacy in medicine. He wrote, “And now that the pharmacists have cloaked even the most nauseous remedies, the temptation is to use medicine on every occasion; and I fear that we may return to the state of polypharmacy, the emancipation from which has been the sole gift of Hahnemann and his followers to the race” [48].

In “Notes on Homeopathy No. 1,” Gates was so enamored with the germ theory that he made this overstated assertion: “The diseases that are now known to be caused by germs are extremely numerous and of the greatest possible variety. They form at least nine-tenths, I should say, of the practice of every physician" (see Appendix). However, in 1900, statistics show that six of the top 10 causes of death in the United States, including heart disease, cerebrovascular disease, kidney disease, accidents, cancer, and senility, were not primarily of infectious origin [49].

In “Notes on Homeopathy No. 3,” Gates clearly demonstrates a profound misunderstanding of homeopathy and of Hahnemann. Gates claimed that Hahnemann had no respect for the wisdom of the body and that the body has no self-healing capacities. Gates wrote, “There is nothing in Dr Hahnemann’s writings to indicate that he supposed nature to be an agency of much, if any, value in the cure of disease” (see Appendix).

However, the underlying principle of homeopathy is a profound respect for the homeostatic mechanism of the body and the mind’s ability to create symptoms as a defense against illness. In fact, homeopathy uses small doses of substances that mimic a sick person’s symptoms because these symptoms serve an inherent defensive function in the body.

In this report, Gates acknowledged the importance of drugs that kill germs, but at the time, he noted that there were only three or four such drugs available. One of the most popular drugs of that time was arsphenamine, also known as Salvarsan or compound 606. It was the first antimicrobial treatment and was commonly used for syphilis, relapsing fever, and African trypanosomiasis. However, once antibiotics began to be used in the 1940s, arsphenamine was widely considered to be a highly dangerous drug that posed significant risks to “life and limb” (literally).

“Notes on Homeopathy No. 4” begins with this statement about homeopathy: “Have patience. The end is in sight.” Gates then reveals his new position: “Scientific medicine is not homeopathy, and it is not allopathy; it is simply scientific” (see Appendix). Here, Gates seems to acknowledge his desire to create a new era of “scientific medicine,” though they did not yet have many successful therapeutic examples of this new medical science, nor did they understand how or why their drugs worked or did not work.

The real paucity of effective medical treatment was acknowledged by the medical leaders of the day, but hidden from the public. In his 1903 presidential address to the AMA, Frank Billings, MD, asserted, “Drugs, with the exception of quinine in malaria and mercury in syphilis, are valueless as cures” [50]. Ironically, even though leading physicians of that time recognized the questionable efficacy and the serious dangers of conventional drugs, they did not convey this information to the public. Instead, they actively and aggressively attacked any alternatives to conventional treatments and anyone who questioned the prevailing medical practices.

In the early 20th century, prominent physicians such as Billings and Osler recognized that very few effective medical treatments were available. However, conventional medicine practitioners of that era rejected any other treatments as quackery and insisted that their approach was the only “scientific medicine.” Although Gates and others claimed that there was no explanation at the time for how homeopathic medicines worked, the same could be said of conventional drugs.

In this report, Gates criticized Hahnemann for having the “ludicrous” belief that the homeopathic “law of similars” was important to healing and questioned the validity of this primary homeopathic principle. Gates also stated that quinine was a “certain” cure in scientific medicine for treating malaria, insisting that “quinine produces neither chills or fever” (see Appendix).

However, Peruvian bark, the source used by drug companies to make quinine, was the first substance Hahnemann tested for its toxicology when he first examined the homeopathic principle of “the law of similars,” as described in Coulter in *Divided Legacy*, page 449 [13]. Because Hahnemann personally experienced a symptom pattern of fever followed by chills, he conjectured that this medicine was effective in treating malaria and other intermittent fevers because it was found to cause them. It has since been confirmed that the side effects of quinine include both chills and fever [51].

Gates maintained an extreme position on homeopathy, asserting, “Dr Hahnemann was absolutely and wholly mistaken both in his theory of cure and in the selection of the drugs which he alleges would cure, and it is perfectly fair to say that no case ever treated by homeopathy was cured by the treatment.” It is unclear on what basis Gates made this statement. In fact, homeopathy gained significant popularity in the United States and Europe due to its impressive successes in treating infectious disease epidemics during the 19th century, including typhoid, cholera, yellow fever, scarlet fever, and pneumonia. To confirm this, a book published in 1900, *The Logic of Figures or Comparative Results of Homeopathic and Other Treatments*, detailed morbidity and mortality rates in American hospitals, penitentiaries, and “insane asylums” [52]. Commonly, the death rate in homeopathic institutions was one-half or even one-eighth of that in conventional medical institutions. These numbers indicate that the death rates in conventional medical institutions were 200% to 800% greater than those in homeopathic institutions.

Gates wrote all the above with a clear bias against homeopathic medicine, championing “scientific medicine” as the emerging medical system. He maintained unflinching support for new “scientific medicine,” even though he privately acknowledged to Rockefeller that only a small number of treatments appeared to be effective.

On May 5, 1911, Gates wrote his "Notes on Homeopathy No. 5," another set of assertions that he did not want to be made public due to concerns that members of the homeopathic community would challenge its accuracy (see Appendix). In this report, he summarizes the incredibly high dilutions used in homeopathy, referring to the quintillionth dose of Nux vomica (the equivalent of 18X or 9C). He equated this dilution to one drop in a canal 1,000 miles long, one mile wide, and 80 feet deep. For extra flourish, Gates added that this amount of water would extend from the Atlantic seaboard to Chicago. Immediately after asserting that homeopathy had never cured a single patient, he wrote, "The fact is that scientific medicine has as yet found cures for only about as many diseases all told as can be numbered on the fingers of the two hands." However, in previous reports on homeopathy, Gates had been even more conservative, acknowledging only four “proven" treatments, making his later statement appear generous.

The problem with this calculation was that Hahnemann and every homeopathic drug manufacturer since his time would need only nine test-tube-sized bottles to make this dilution, totaling just nine test tubes of water. This dose is substantially smaller than the one Gates suggested so vividly but inaccurately.

Bias and Influence in Rockefeller’s Philanthropic Policies

Why did Rockefeller choose not to support homeopathic institutions in the 20th century? This question cannot be easily answered, as J. D. Rockefeller left no correspondence on this subject. However, one noted historian specializing in homeopathic history asserted that Rockefeller “was dissatisfied at the inability of the homeopathic institutions to teach and promulgate the Hahnemannian doctrines.”

Only a relatively small number of homeopathic physicians were considered ardent practitioners of Hahnemannian or “classical” homeopathy. Such adherence to the original homeopathic methodology required highly individualized treatment. Most homeopathic physicians, however, used various shortcuts to select homeopathic medicines based more on the disease the person had and on simple keynote prescribing, rather than on the more laborious process of prescribing based on the totality of a sick person’s syndrome. This shortcut method resembled the conventional medical model of prescribing medicines based on diagnosed diseases. It did not follow the principles of classical homeopathy, which individualized treatment for each patient based on the person's entire physical and psychological syndrome, of which the diagnosed disease was only one part.

In fact, this small group of homeopaths broke away from the American Institute of Homeopathy to create the International Hahnemannian Association, as described by Chernow in Titan, page 370 [1]. It is important for historians to recognize that there have always been different schools of thought and practice in homeopathy. Understanding this nuance is essential, and Rockefeller appreciated the homeopathic school of thought and practice that used the most dilute and potentized medicines, the type of practice that most infuriated and confused critics of homeopathy.

Gates’s reports on homeopathy may have played into this concern by convincing Rockefeller to believe that “scientific medicine” was the future of medical care. Gates also surrounded Rockefeller with respected medical and scientifically trained individuals who supported this view. However, Gates cannot be solely blamed for the lack of support for homeopathy, as Rockefeller was allowed to allocate $2 million per year to his pet projects until at least 1917, as described by Chernow in *Titan*, page 479 [1].

Another element that may explain the lack of funding for homeopathic institutions was the Rockefeller Foundation’s policy of avoiding subjects that “smacked of controversy,” and homeopathic medicine certainly fell into this category, as described by Chernow in *Titan*, page 568 [1].

Perhaps the strongest explanation for the Foundation’s avoidance of support for homeopathic institutions was the influence of Rockefeller’s son, Junior. He joined the Rockefeller philanthropic efforts around the same time Gates wrote his reports on homeopathy and served as the Foundation’s president from 1913 until 1934.

One emblematic story of how Junior shut down efforts by homeopaths to secure funding involves Julia Minerva Green, MD (1871-1963), a co-founder of the American Foundation for Homeopathy, and Mary Coffin Ware Dennett (1872-1947), a prominent women’s rights activist and pioneer in birth control, sex education, and women’s suffrage, as well as a vociferous advocate for homeopathy. When Green and Dennett contacted Junior’s secretary, W. H. Richardson, they encountered significant resistance. Richardson clarified that Junior would not support their cause, stating that he would “have none of it.”

By 1926, when Dennett and Green sought to make a more concerted effort to secure financial support for homeopathy, they turned to Dr. Hamilton Biggar, Rockefeller’s closest friend and long-time personal homeopath, for advocacy. Unfortunately, Biggar had recently passed away.

In 1917, Junior persuaded his father to contribute an additional $50 million to the RIMR. To advocate for supporting conventional medical projects, he argued, “This is a field in which there can be no controversy so that I think the possibility of criticism as regards the use of the fund or its potential dangers would be almost nothing.”

Moreover, Rockefeller’s biographer, Ron Chernow, claims that the Rockefeller Foundation used science as “the magic wand waved over any project to show that it was sound and objective, free of favoritism or self-interest.” Chernow also asserts that, for a time, the foundations avoided funding projects in the humanities and social sciences, considering these areas “too subjective or fraught with political peril.”

Nonetheless, several factors involved in this matter are often overlooked but should not be disregarded.

When B. C. Forbes, the founder of Forbes magazine, asked Rockefeller to name the greatest businessman he had ever encountered, Rockefeller did not name Henry Ford or Andrew Carnegie. Instead, he claimed it was the head of his foundations, Frederick Gates, as described by Chernow in *Titan*, page 370 [1].

This insight may explain how and why Gates was so successful in persuading Rockefeller to support “scientific medicine” over homeopathic medicine. Many of the most popular conventional medical drugs of the day were coal tar derivatives, products derived from Rockefeller’s oil refineries. For instance, the starting material for the chemical synthesis of aspirin is benzene, derived from petroleum. Similarly, acetaminophen, discovered in 1878, originated from crude oil. Additionally, numerous ointments, shampoos, and soaps were coal tar derivatives, providing antifungal, anti-inflammatory, anti-itch, and antiparasitic properties.

Gates’s business acumen was so compelling, or at least financially beneficial to the Rockefeller enterprises, that it prevented any interference with or alterations to their pro-scientific medicine initiatives.

During the first five decades of the 20th century, Rockefeller's philanthropy, led by J. D. Rockefeller and his son, donated over $1 billion. Despite this substantial generosity, there is no public record indicating that John D. Rockefeller was aware that none of the funds from his philanthropic foundations supported any homeopathic institutions. “Through his philanthropies, Rockefeller did more than anyone else to destroy homeopathy in America, and in the end, he seemed powerless to stop the scientific revolution that he himself had so largely set in motion,” as described by Chernow in *Titan*, page 479 [1]. However, given Rockefeller’s reputation for meticulous accounting and comprehensive knowledge of financial matters, it seems unlikely that he was unaware of how his foundations were allocating funds to these institutions.

Discussion

While this analysis draws on extensive documentation, the absence of direct correspondence from Rockefeller himself limits definitive conclusions about his personal intent.

Perhaps the findings presented here will inspire other researchers to investigate further why the Rockefeller Foundation ignored his persistent requests to support homeopathic institutions.

This historical analysis reveals the significant role that private influence and institutional power played in shaping the trajectory of American medical education. Despite John D. Rockefeller’s personal reliance on and respect for homeopathic physicians, his philanthropic foundations, heavily guided by the views of his adviser Frederick T. Gates, actively withheld support from homeopathic medical institutions. Gates’s unpublished reports, marked by ideological bias and factual inaccuracies, had a disproportionate impact on decisions that would shape public health infrastructure for decades.

These findings complicate the common narrative that the Flexner Report’s recommendations were adopted purely in the interest of science or public health. The collaboration between the AMA, Abraham Flexner, and the Rockefeller Foundation highlights the interplay between personal beliefs, institutional strategy, and public policy. Additionally, the AMA’s growing financial dependence on advertising revenue during this time adds another layer of commercial motivation that has received little attention in historical literature.

This work underscores the importance of reexamining medical history through a lens that accounts not only for scientific advancement but also for the underlying political, ideological, and economic forces at play. Doing so can help us better understand how certain systems of medicine, such as homeopathy, were marginalized, and how the legacies of those decisions continue to shape public perception and policy today.

These reflections pave the way for a broader reconsideration of how financial power, institutional loyalty, and ideological convictions shaped modern medical history.

With the emergence of the Industrial Revolution and the cultural drive toward America as a “melting pot,” the nineteenth century saw increasing homogenization in medical care and a growing assumption that there was only one “right” method to heal. Yet the United States has also long harbored a countervailing desire for medical pluralism and cultural diversity in health care. This historical tension resonates with contemporary debates about how dominant paradigms are shaped, not only by scientific discovery but also by economic power, political ideology, and cultural forces.

By drawing these connections forward, this study encourages reflection on how institutional alliances and philanthropic strategies continue to shape which modalities are considered legitimate in American medicine. While the early 20th century saw homeopathy marginalized, today other integrative or nonconventional approaches face similar scrutiny. Acknowledging this pattern may help foster more inclusive frameworks that allow scientific medicine and traditional or complementary practices to coexist in ways that better reflect the pluralistic values of modern society.

## Conclusions

The prevailing historiographical understanding of how “scientific medicine” became dominant often attributes its success to therapies deemed more effective than those of its leading competitor, homeopathy. However, this analysis reveals that J. D. Rockefeller’s immense wealth played a critical role in elevating scientific medicine as the dominant force, despite his personal reliance on homeopathic physicians and repeated indications of support for homeopathic institutions. Through five private reports prepared by Frederick T. Gates, Rockefeller’s business manager and chief philanthropic adviser, a previously unexamined chapter in medical history is brought to light. These documents reflect Gates’s deep bias against homeopathy and admiration for William Osler, ironically, a physician who respected homeopathy and its founder, Samuel Hahnemann.

Although modest support for homeopathy existed in the late 19th century, it disappeared entirely in the 20th century. Rockefeller’s philanthropic institutions, under Gates’s influence, withheld funding from homeopathic colleges and hospitals, forcing them to abandon their foundational principles or close entirely. While homeopathy was largely erased from American medical education, its continued use in Europe, Asia, and South America suggests it retains therapeutic relevance. This is not to claim that homeopathy should dominate medical care, but it may merit renewed consideration within a more integrative and collaborative framework for health and medicine.

## Figures and Tables

**Figure 1:**
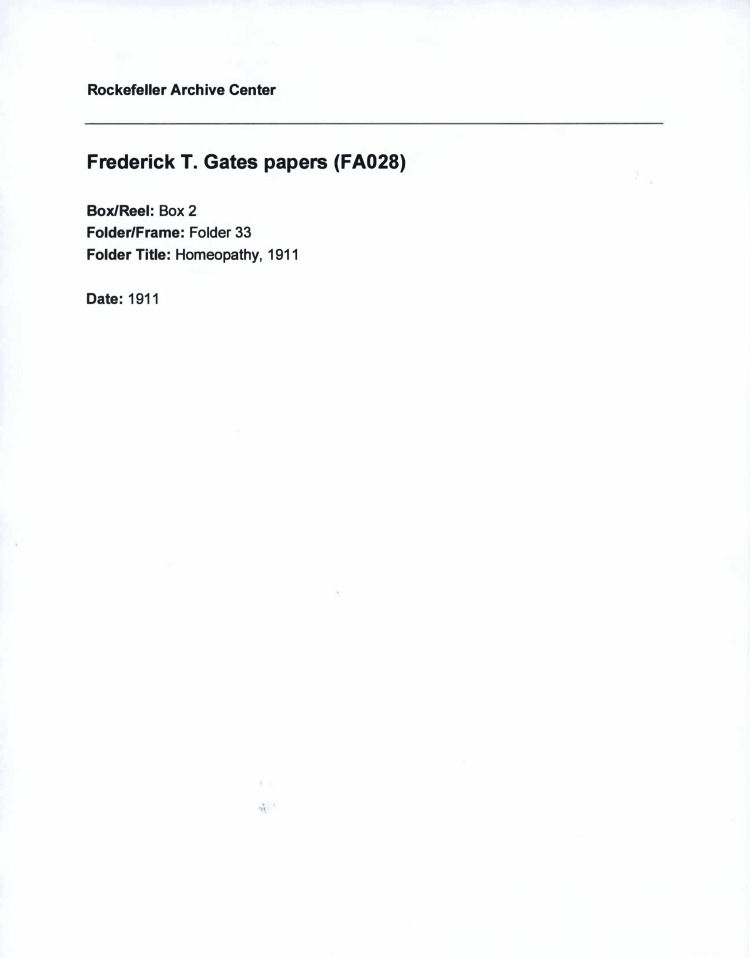
Page 1

**Figure 2:**
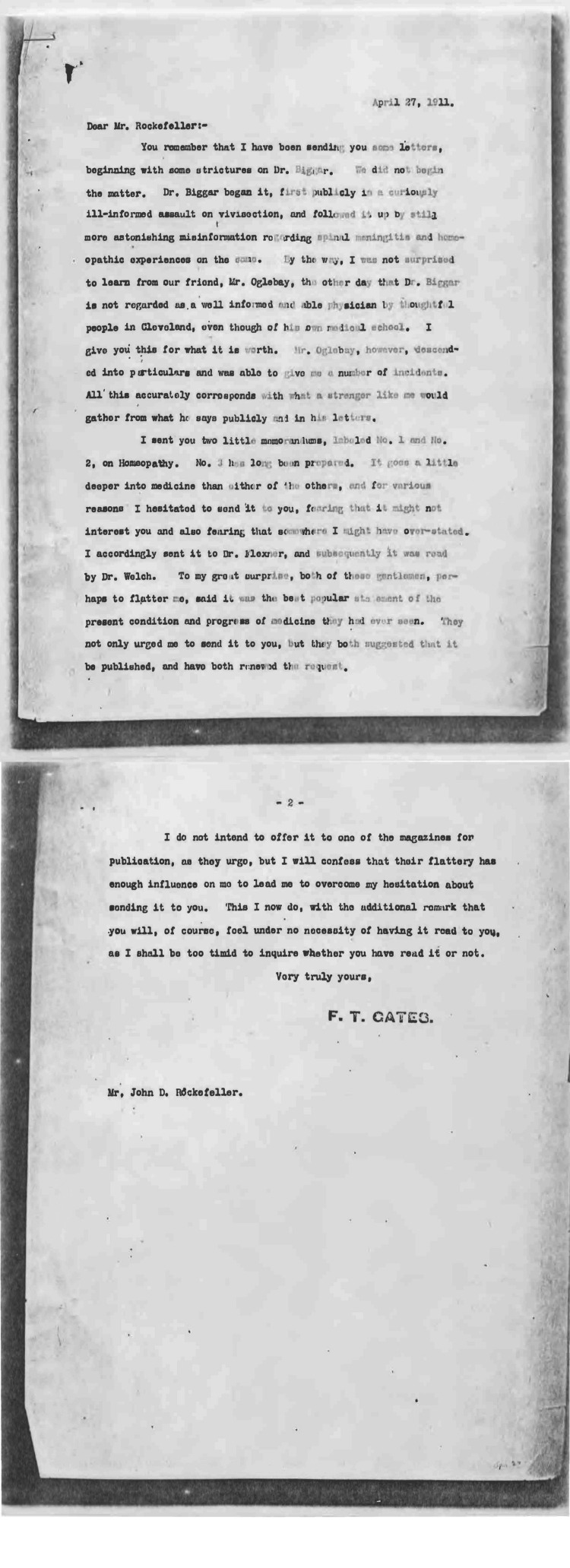
Page 2

**Figure 3:**
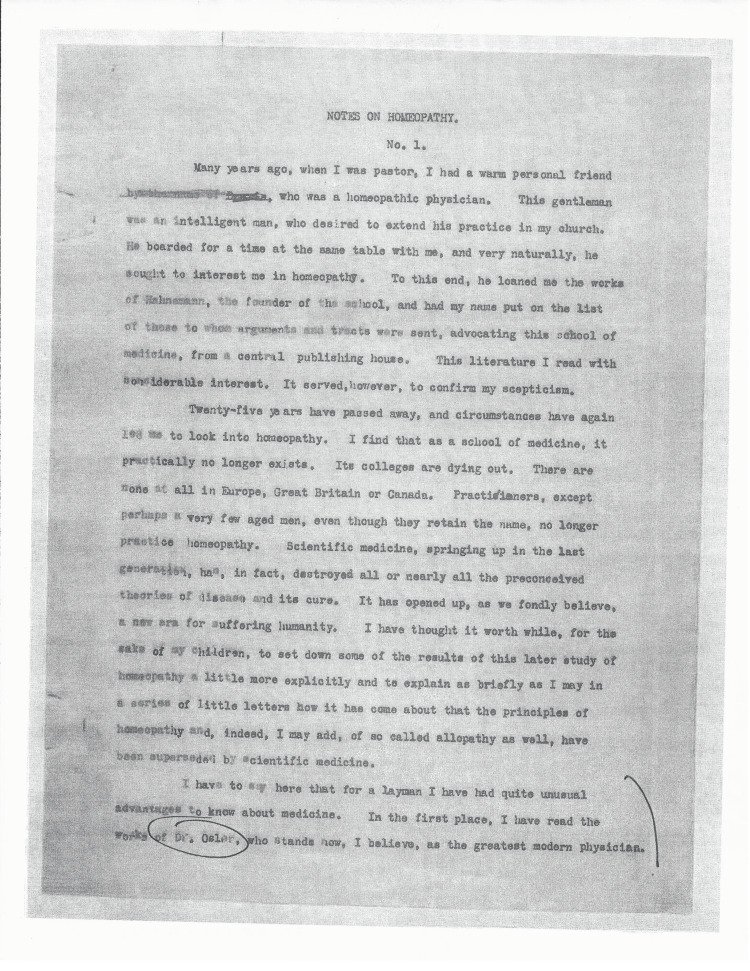
Page 3 - Report #1

**Figure 4:**
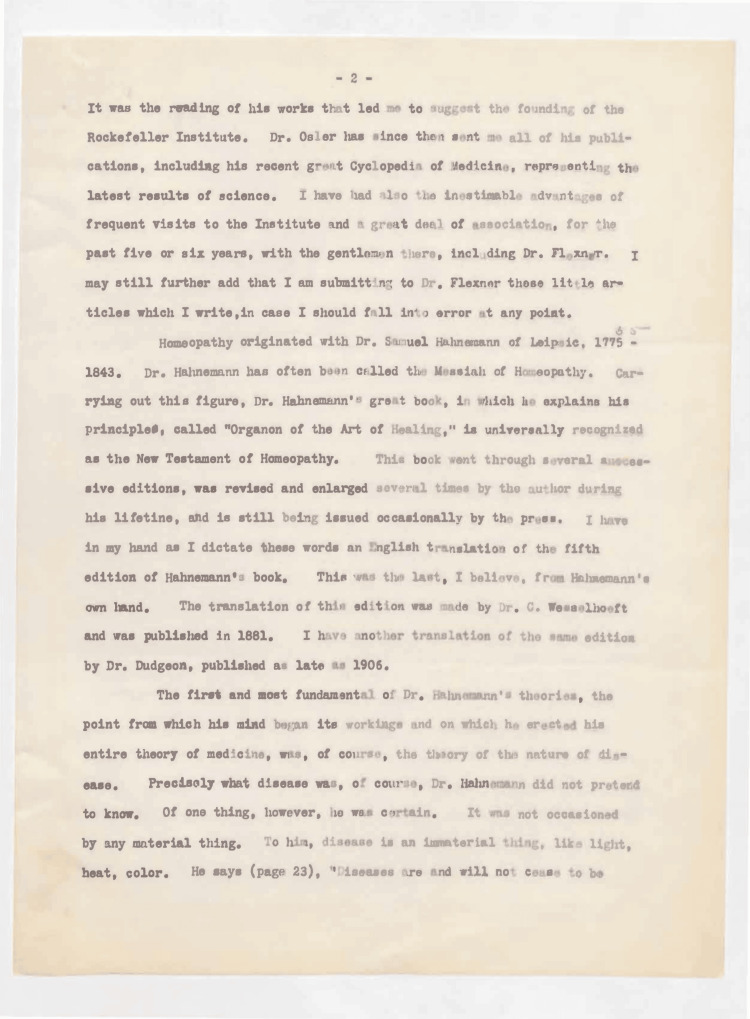
Page 4

**Figure 5:**
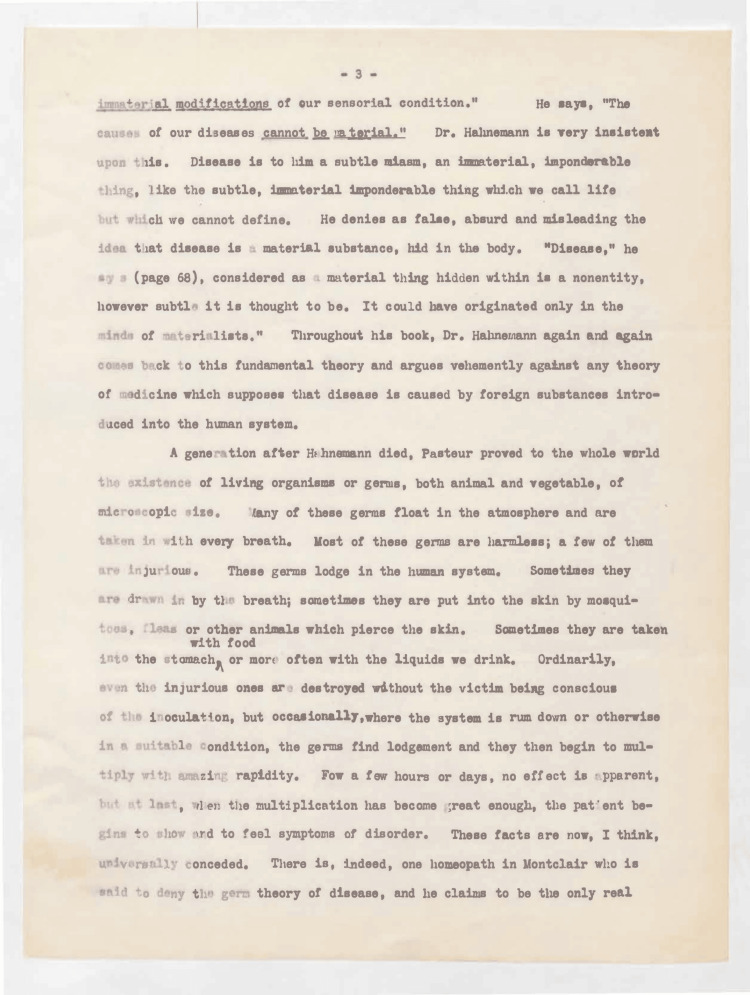
Page 5

**Figure 6:**
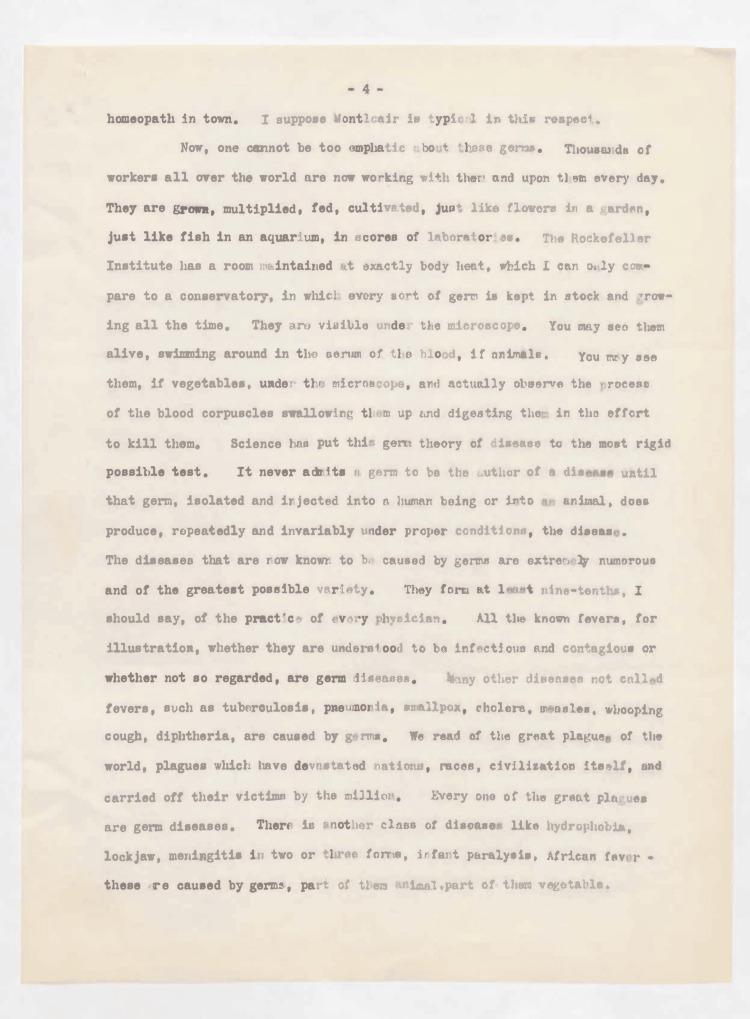
Page 6

**Figure 7:**
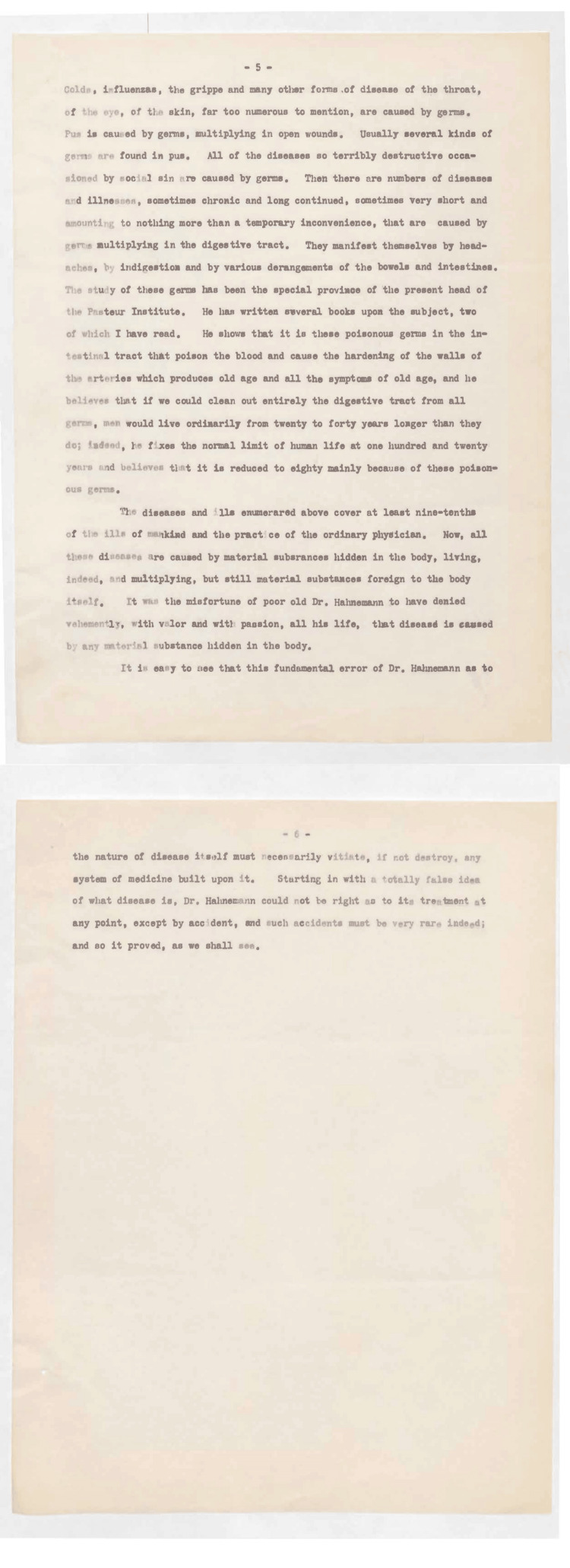
Page 7

**Figure 8:**
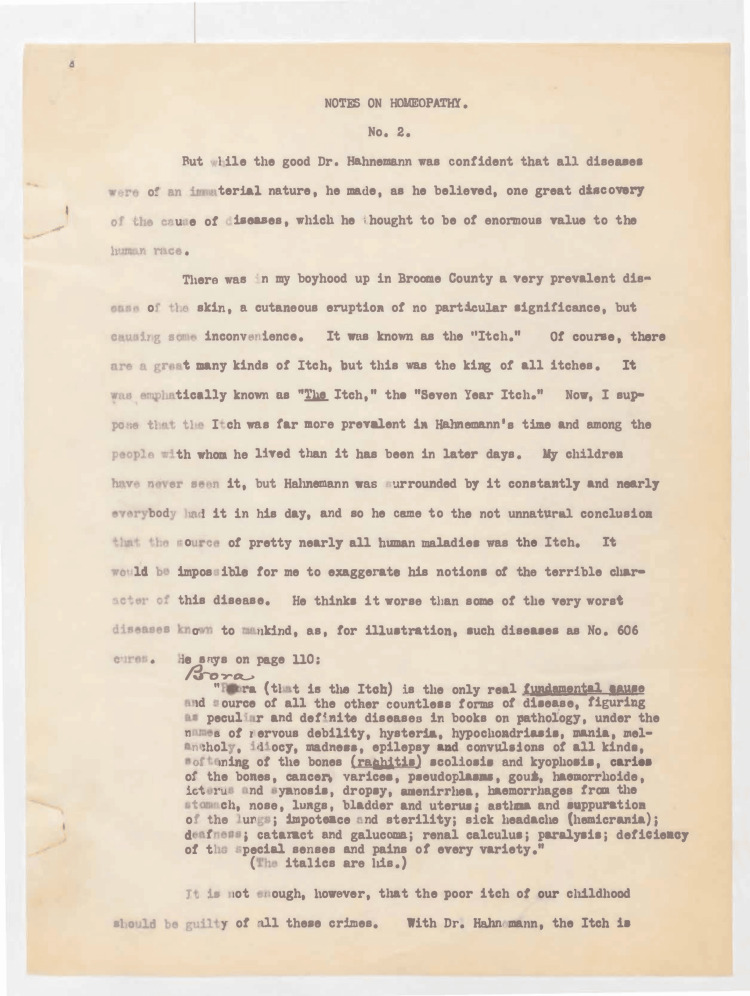
Page 8 - Report #2

**Figure 9:**
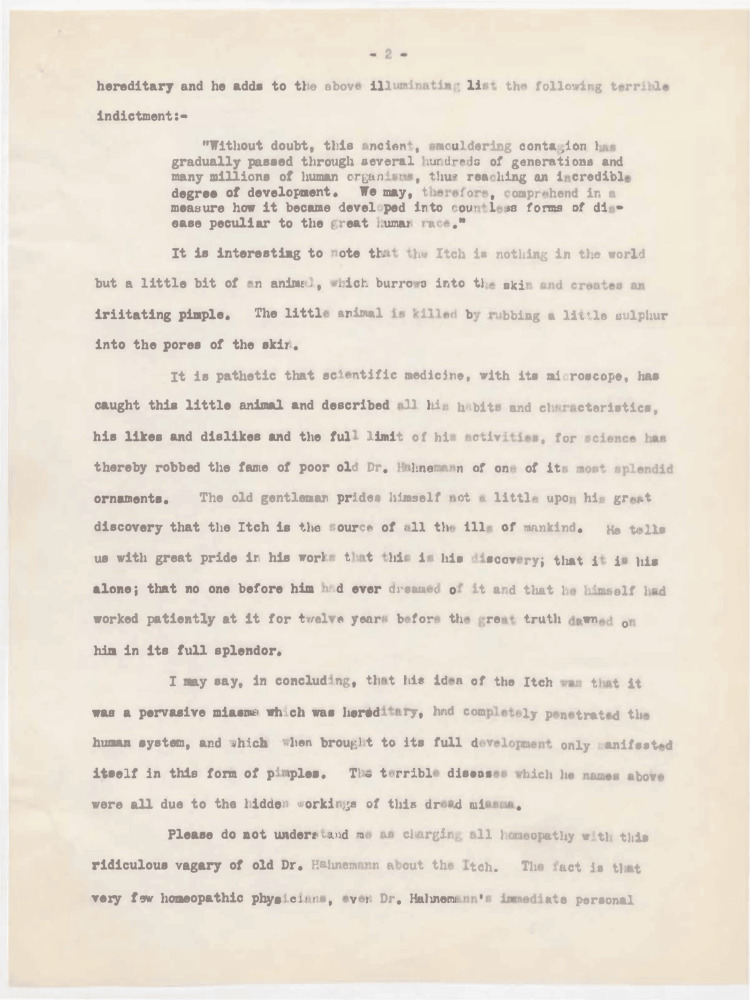
Page 9

**Figure 10:**
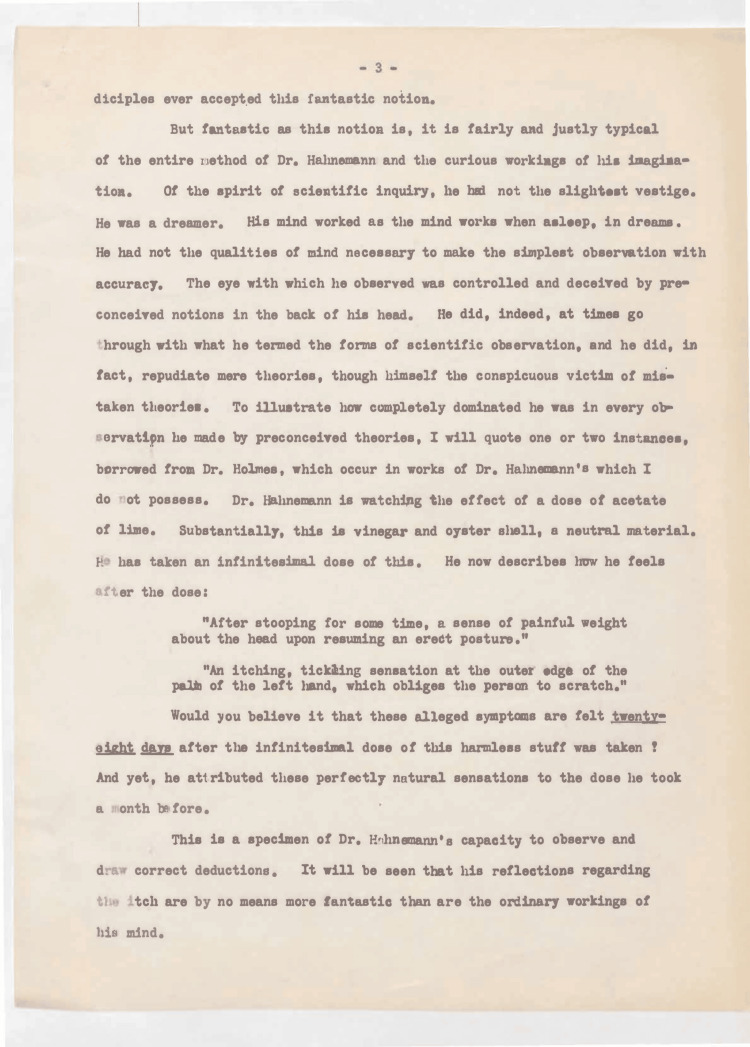
Page 10

**Figure 11:**
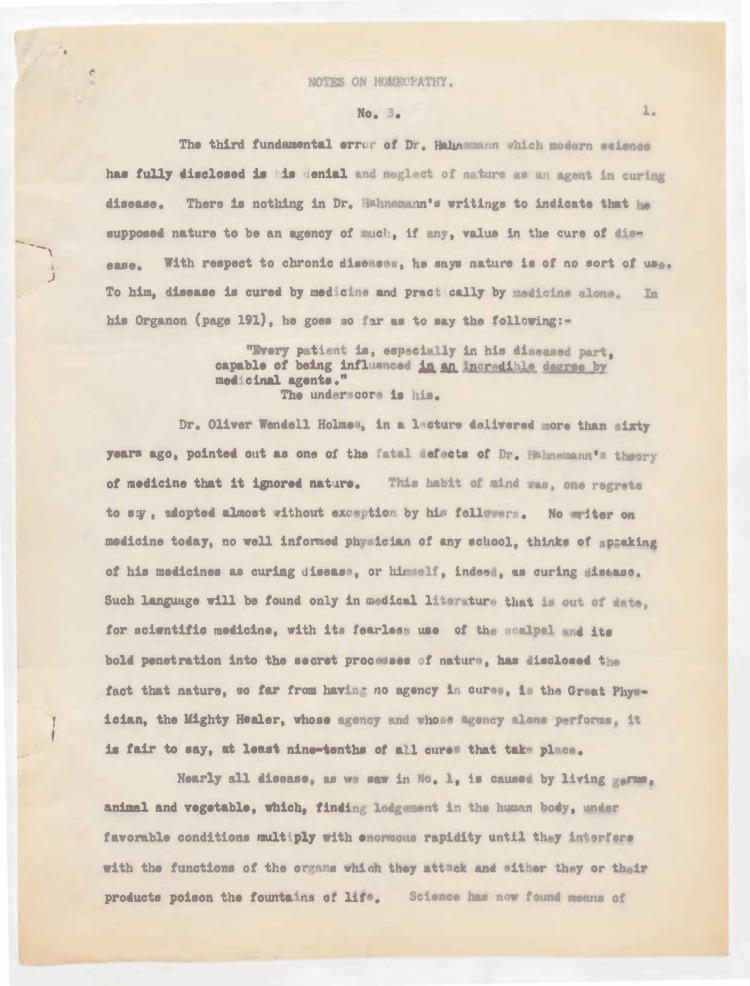
Page 11 - Report #3

**Figure 12:**
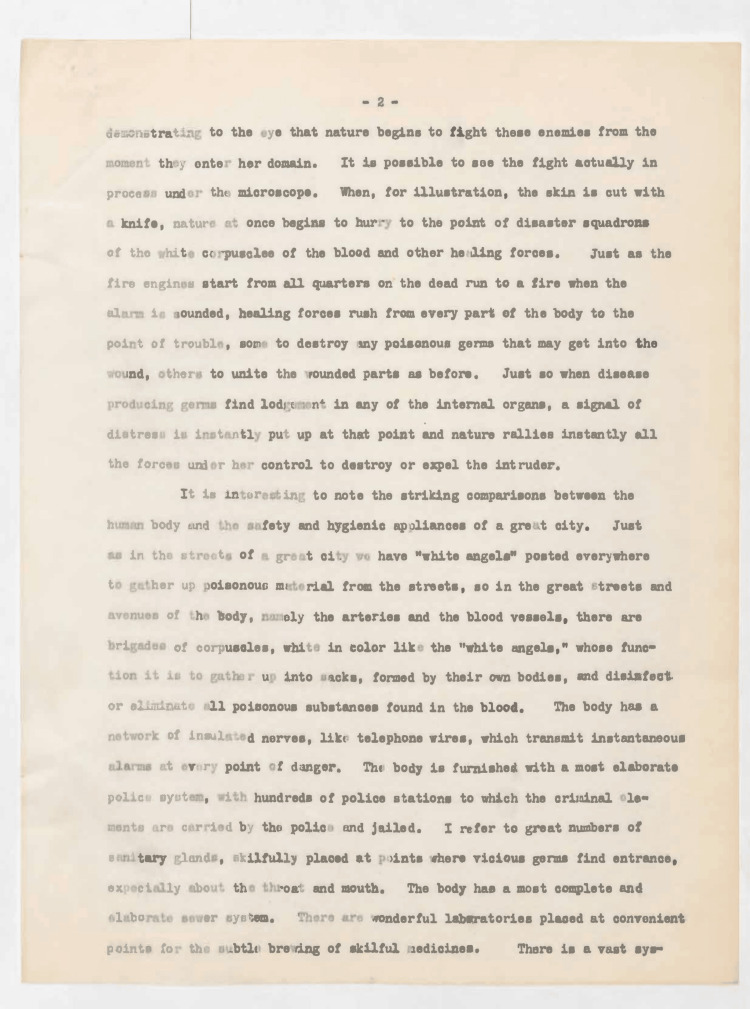
Page 12

**Figure 13:**
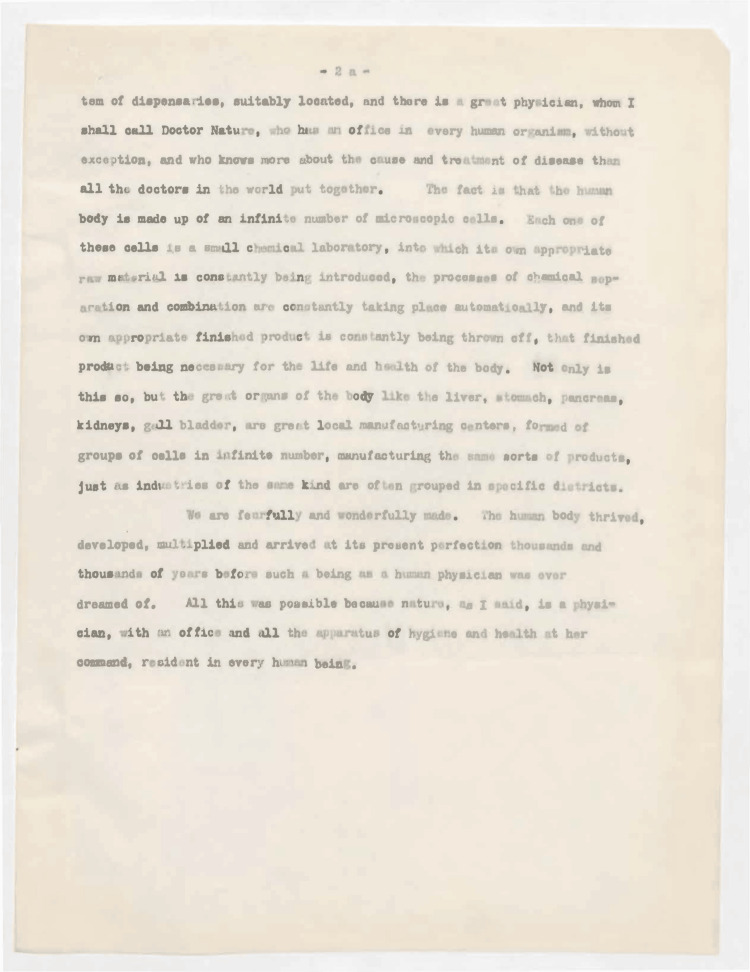
Page 13

**Figure 14:**
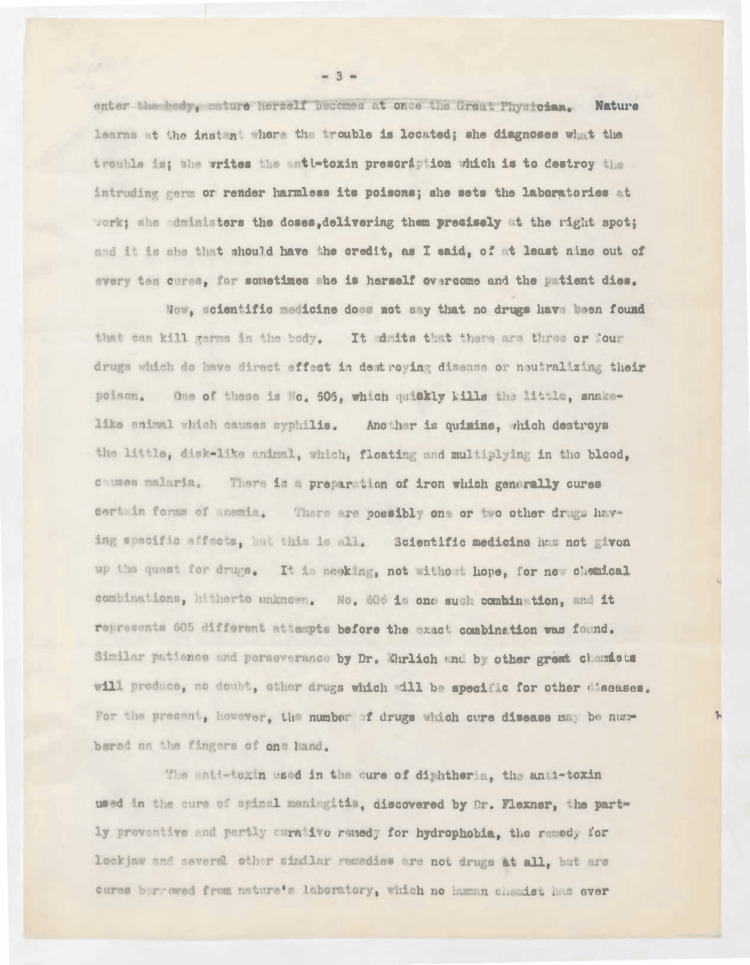
Page 14

**Figure 15:**
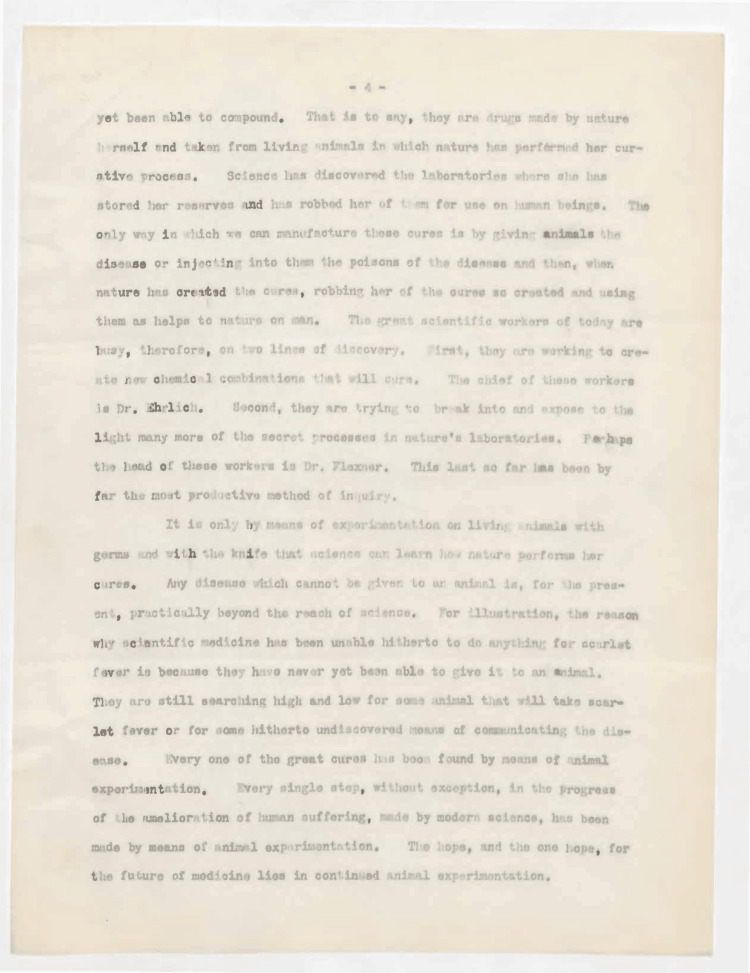
Page 15

**Figure 16:**
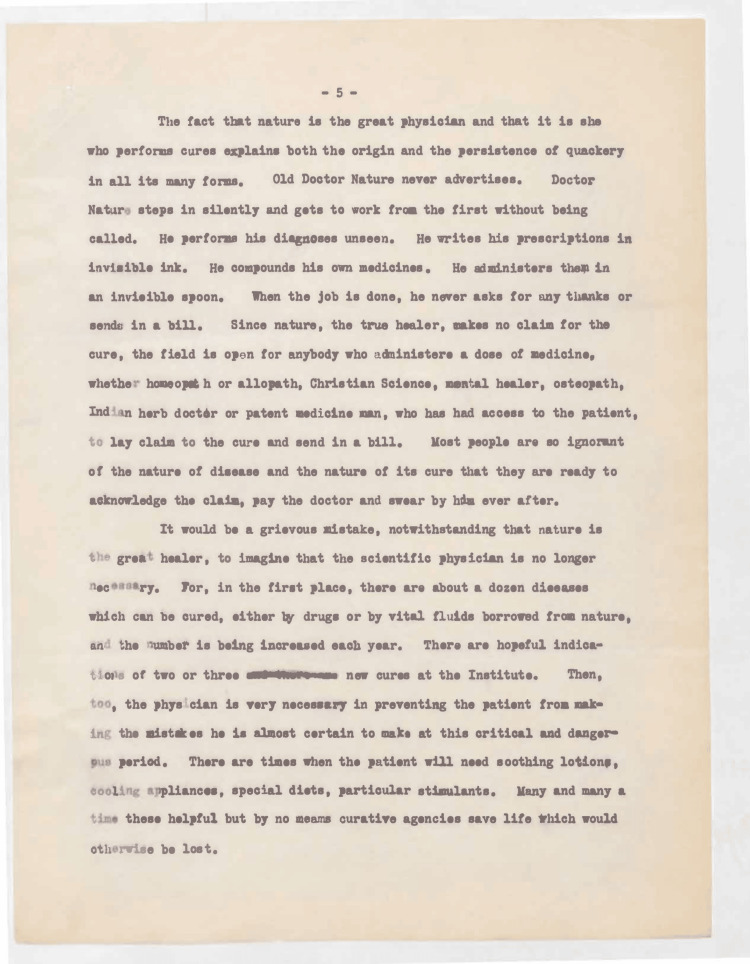
Page 16

**Figure 17:**
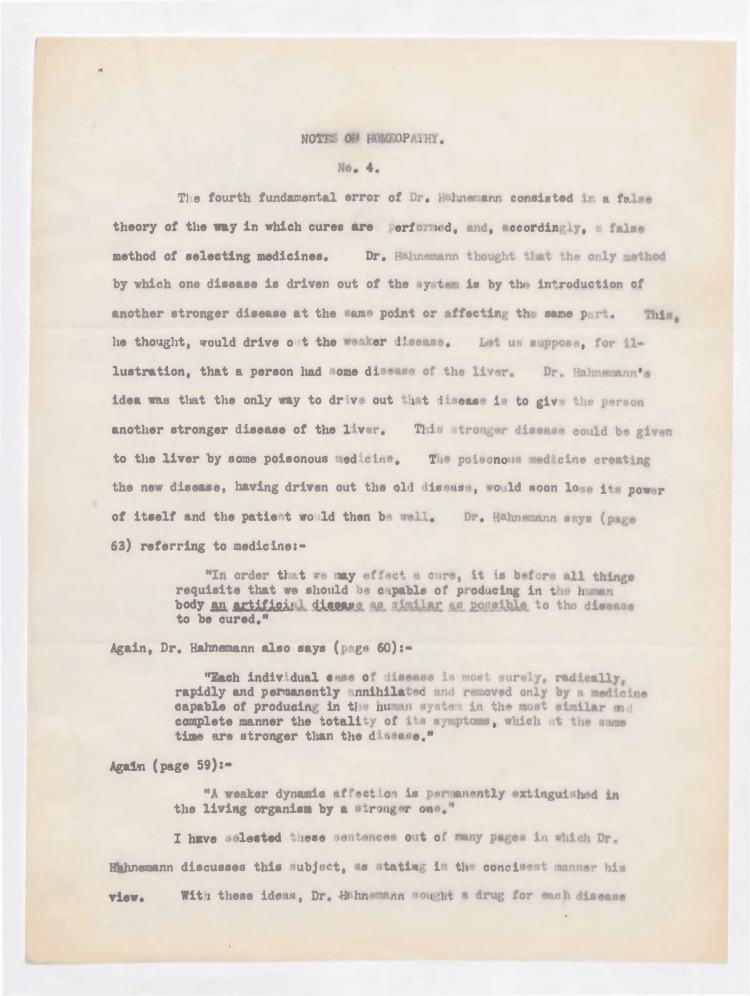
Page 17 - Report #4

**Figure 18:**
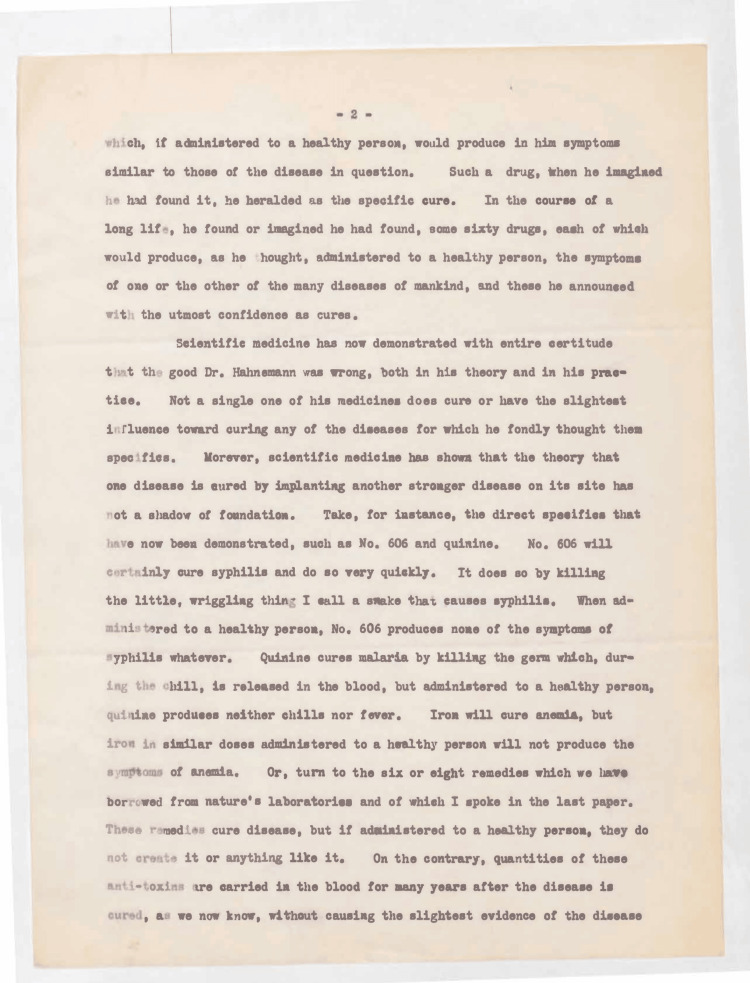
Page 18

**Figure 19:**
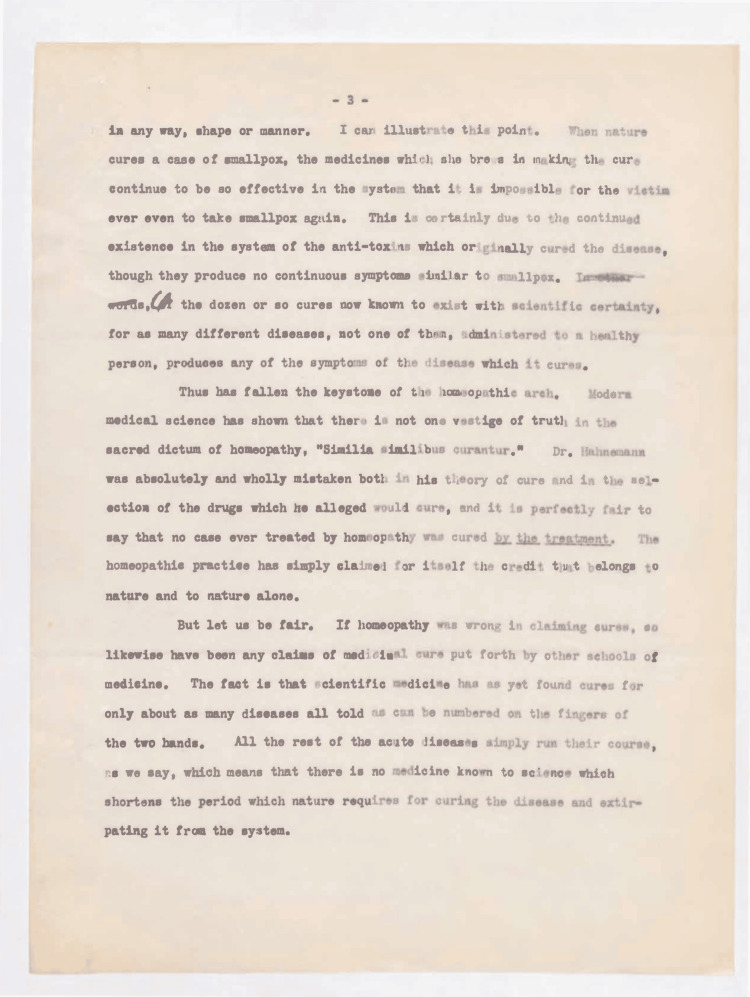
Page 19

**Figure 20:**
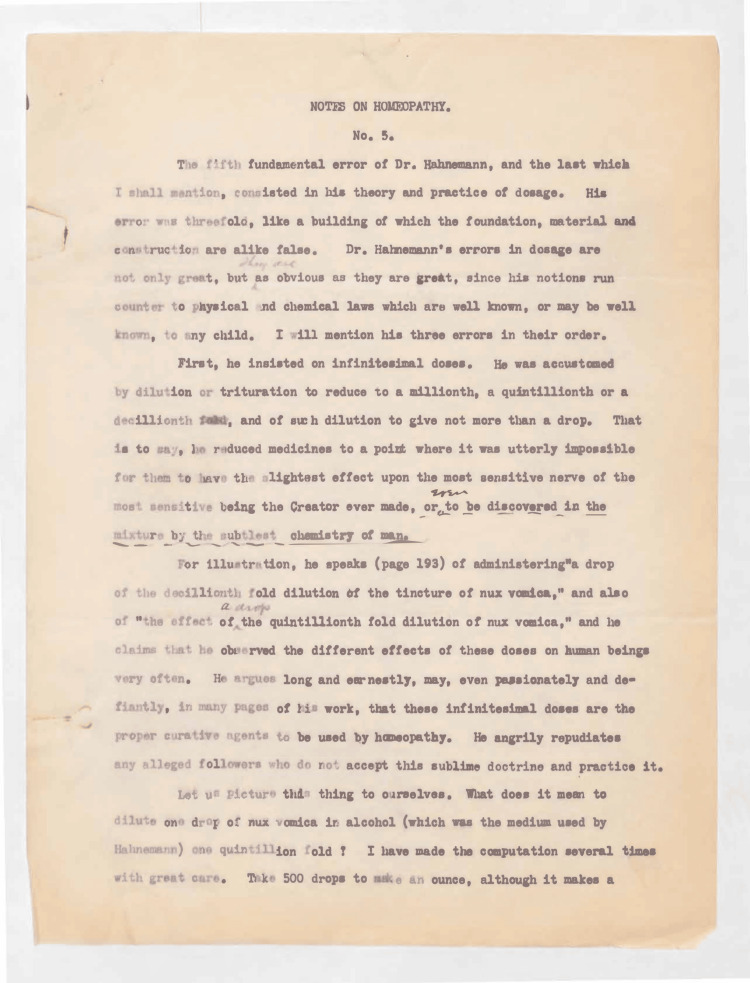
Page 20 - Report #5

**Figure 21:**
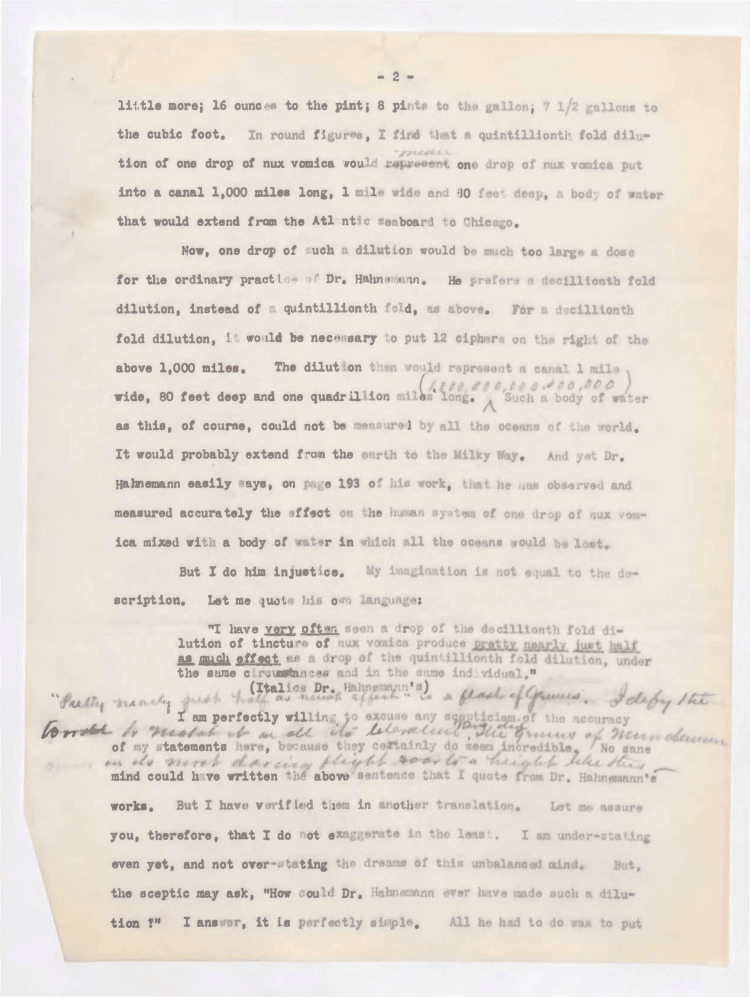
Page 21

**Figure 22:**
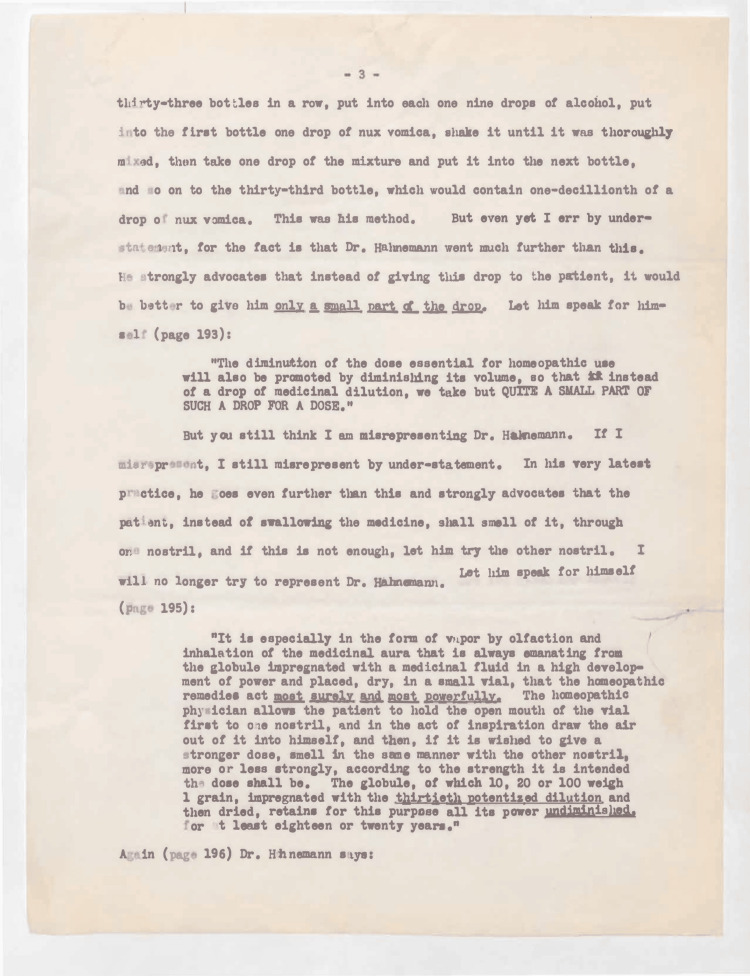
Page 22

**Figure 23:**
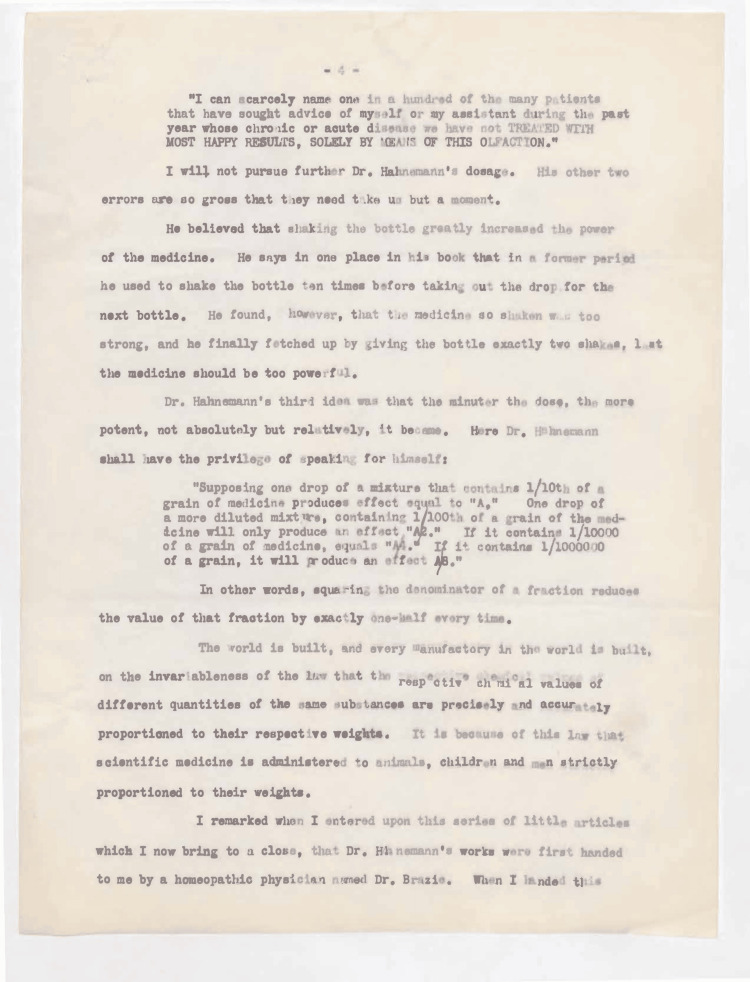
Page 23

**Figure 24:**
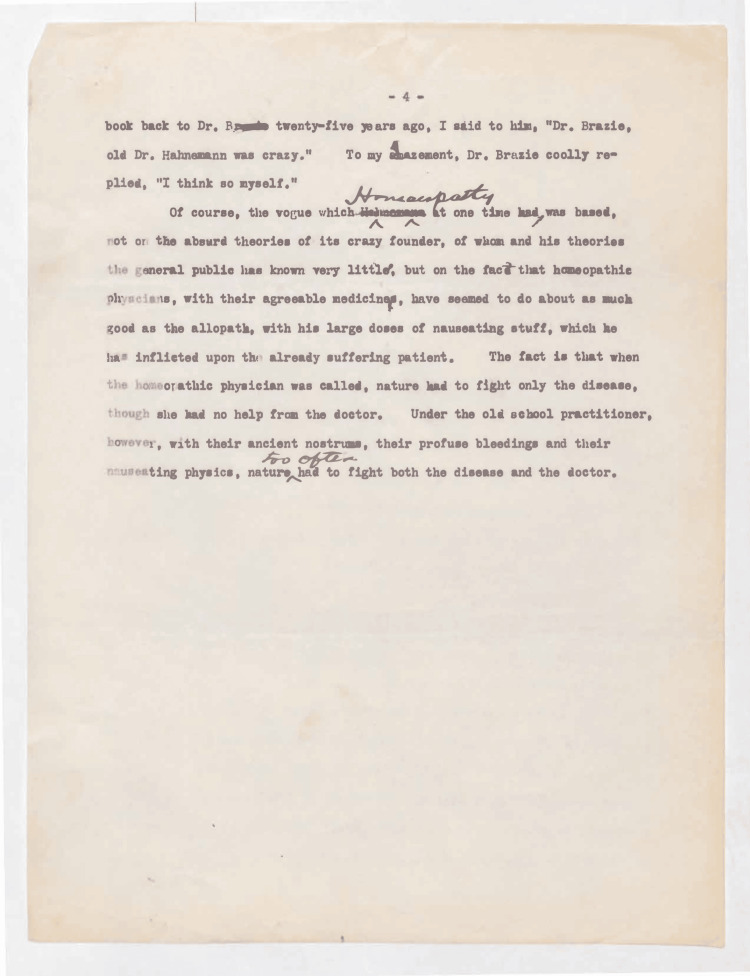
Page 24

**Figure 25:**
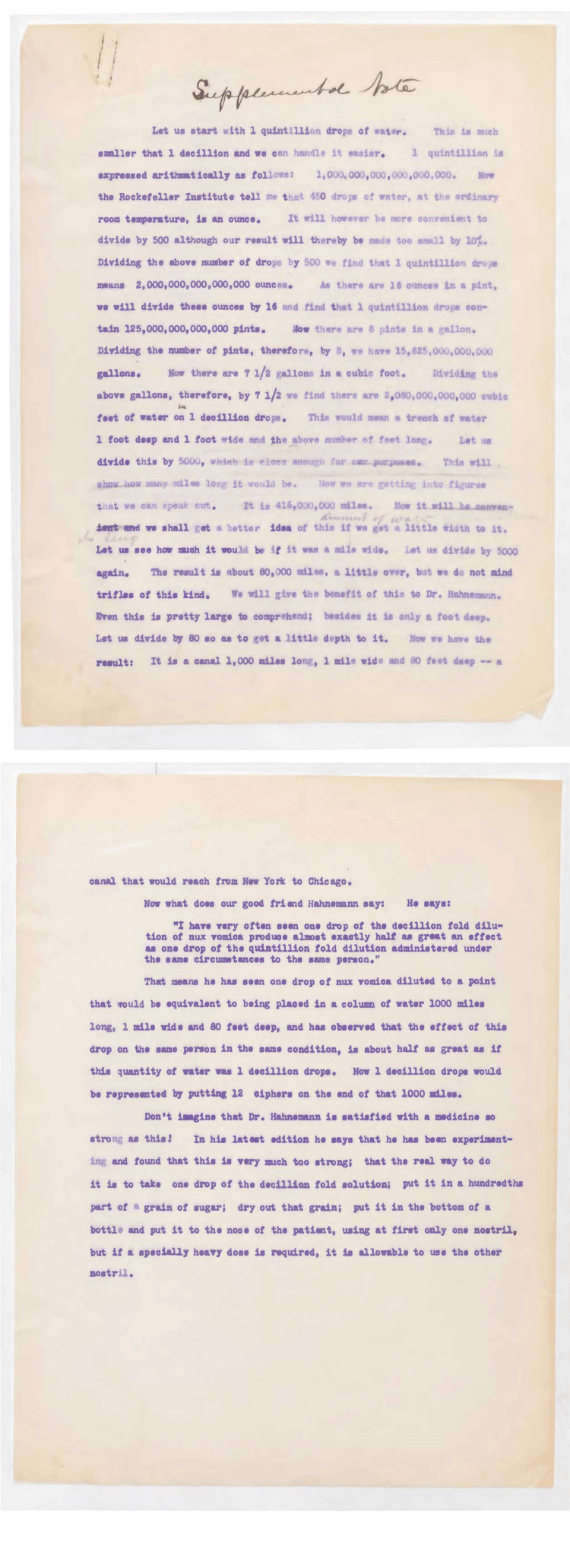
Page 25 - Supplemental Note

## Appendices

Rockefeller Archive Center: Frederick T. Gates papers (FA028) (https://drive.google.com/file/d/12gWRUSaLLeeze270hNoiUMqoWdHzovJu/view).
